# Role of Single-Nucleotide Polymorphisms in Genes Implicated in Capecitabine Pharmacodynamics on the Effectiveness of Adjuvant Therapy in Colorectal Cancer

**DOI:** 10.3390/ijms25010104

**Published:** 2023-12-20

**Authors:** Yasmin Cura, Almudena Sánchez-Martín, Noelia Márquez-Pete, Encarnación González-Flores, Fernando Martínez-Martínez, Cristina Pérez-Ramírez, Alberto Jiménez-Morales

**Affiliations:** 1 Pharmacy Service, Pharmacogenetics Unit, University Hospital Virgen de las Nieves, 18014 Granada, Spain; e.yacura@go.ugr.es (Y.C.);; 2 Medical Oncology, University Hospital Virgen de las Nieves, 18014 Granada, Spain; 3 Biomedical Research Institute—ibs.Granada, 18012 Granada, Spain; 4 Pharmaceutical Care Research Group, Pharmacy Faculty, University of Granada, 18016 Granada, Spain; 5 Department of Biochemistry and Molecular Biology II, Institute of Nutrition and Food Technology “José Mataix”, Center of Biomedical Research, University of Granada, 18016 Granada, Spain

**Keywords:** colorectal cancer, capecitabine, pharmacodynamics, single nucleotide polymorphisms, survival

## Abstract

Colorectal cancer (CRC) is a highly prevalent form of neoplasm worldwide. Capecitabine, an oral antimetabolite, is widely used for CRC treatment; however, there exists substantial variation in individual therapy response. This may be due to genetic variations in genes involved in capecitabine pharmacodynamics (PD). In this study, we investigated the role of single-nucleotide polymorphisms (SNPs) related to capecitabine’s PD on disease-free survival (DFS) in CRC patients under adjuvant treatment. Thirteen SNPs in the *TYMS*, *ENOSF1*, *MTHFR*, *ERCC1*/*2*, and *XRCC1*/*3* genes were genotyped in 142 CRC patients using real-time PCR with predesigned TaqMan^®^ probes. A significant association was found between favorable DFS and the *ENOSF1* rs2612091-T allele (*p* = 0.010; HR = 0.34; 95% CI = 0.14–0.83), as well as with the *TYMS*/*ENOSF1* region ACT haplotype (*p* = 0.012; HR = 0.37; 95% CI = 0.17–0.80). Other factors such as low histological grade (*p* = 0.009; HR = 0.34; 95% CI = 0.14–0.79) and a family history of cancer (*p* = 0.040; HR = 0.48; 95% CI = 0.23–0.99) were also linked to improved DFS. Therefore, the SNP *ENOSF1* rs2612091 could be considered as a predictive genetic biomarker for survival in CRC patients receiving capecitabine-based adjuvant regimens.

## 1. Introduction

Colorectal cancer (CRC) is one of the most frequently diagnosed neoplasms and is considered one of the leading causes of cancer-related morbidity and mortality worldwide [1]. In the United States, CRC has a significant impact, affecting approximately 135,439 new patients annually and standing as the second leading cause of cancer-related mortality, with approximately 50,260 deaths each year [2,3]. Adenocarcinomas (ADC) constitute the majority of CRC, representing 90% of all cases, while other histological types, such as adenosquamous, spindle, squamous, and undifferentiated, are less frequently observed [2,3]. The approach to CRC treatment is comprehensive and individualized based on patient and tumor characteristics. Therapeutic strategies encompass both local and systemic approaches. Local strategies involve surgical resection or endoscopic resection, the latter being particularly employed for favorable-risk and early-stage disease when identified in polyps. Systemic approaches include chemotherapy, targeted therapy, and immunotherapy. Chemotherapy encompasses various drug options, such as fluoropyrimidines (FP), which include 5-fluorouracil (5-FU), capecitabine, S-1, tegafur plus uracil, and other antineoplastic agents like irinotecan, trifluridine/tipiracil, and oxaliplatin. The administration of chemotherapy can be tailored to different settings, including neoadjuvant, adjuvant, metastatic, or palliative, aligning with the specific needs of each patient [2,3]. While surgery stands as the standard treatment for patients with localized-stage CRC, those in advanced stages who are eligible for surgery typically undergo adjuvant chemotherapy or radiotherapy [2,3]. Capecitabine, an orally administered prodrug of the antimetabolite agent 5-FU belonging to the FP family, is a standard element within adjuvant chemotherapy regimens used in CRC therapy [4]. Nevertheless, high interindividual variability in treatment response is observed among patients with CRC who are treated with capecitabine-based regimens [5]. Pharmacogenetics has emerged as a promising field to elucidate genetic determinants that contribute to interpatient variation in drug response [6]. In fact, genotyping of variants in the dihydropyrimidine dehydrogenase (*DPYD*) gene before initiating FP therapy has become a standard practice in numerous hospitals and is considered a valuable tool for predicting severe FP toxicity. Nonetheless, it is important to note that, currently, there are no established genetic biomarkers for predicting capecitabine-based therapy effectiveness.

**Figure 1:**
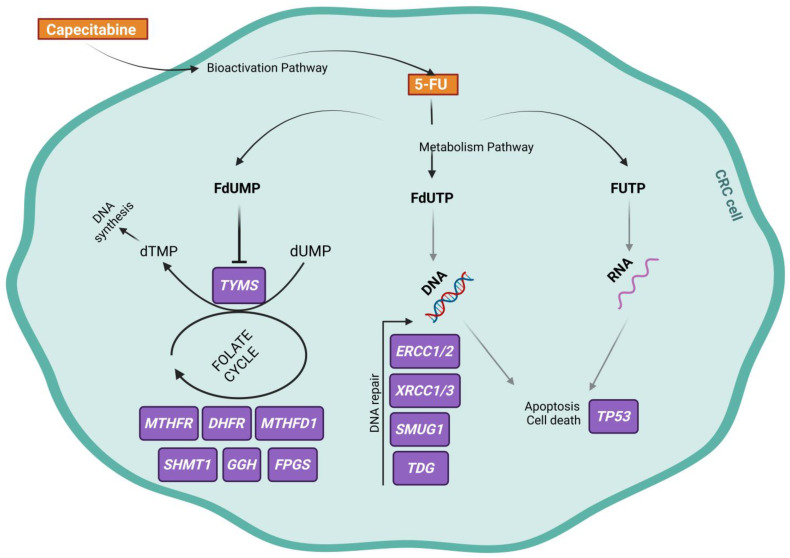
Capecitabine pharmacodynamics pathway. Created with BioRender.com. 5-FU: fluorouracil; CRC: colorectal cancer; dTMP: 2′-deoxythymidine-5′-monophosphate; dUMP: 2′-deoxyridine-5′-monophosphate; DHFR: dihydrofolate reductase; *ERCC1*: ERCC excision repair 1, endonuclease non-catalytic subunit; *ERCC2*: ERCC excision repair 2, TFIIH core complex helicase subunit; *ENOSF1*: enolase superfamily member 1; FdUMP: fluorodeoxyuridine monophosphate; FdUTP: fluorodeoxyuridine triphosphate; FPGS: folylpolyglutamate synthase; FUTP: fluorouridine triphosphate; GGH: gamma-glutamyl hydrolase; MTHFD1: methylenetetrahydrofolate dehydrogenase; *MTHFR*: methylenetetrahydrofolate reductase; SHMT1: serine hydroxy methyltransferase 1; SMUG1: single-strand-selective monofunctional uracil-DNA glycosylase 1; SNP: single-nucleotide polymorphism; TDG: thymine DNA glycosylase; TP53: tumor protein P53; *TYMS*: thymidylate synthetase; *XRCC1*: X-ray repair cross-complementing 1; *XRCC3*: X-ray repair cross-complementing 3.

Single-nucleotide polymorphisms (SNPs) in genes encoding proteins involved in the pharmacodynamics (PD) pathway of capecitabine have the potential to influence treatment outcomes and drug survival [7]. As a prodrug, capecitabine undergoes enzymatic bioactivation to its active form 5-FU. The active drug is subsequently metabolized into three pharmacologically active metabolites: fluorodeoxyuridine monophosphate (FdUMP), fluorouridine triphosphate (FUTP), and fluorodeoxyuridine triphosphate (FdUTP). FdUMP acts as an inhibitor of thymidylate synthase (TS), an enzyme encoded by the thymidylate synthase gene (*TYMS*), which plays a crucial role in pyrimidine and DNA synthesis. Consequently, this inhibition disrupts the conversion of 5,10-methylenetetrahydrofolate (5,10-MTHF) to dihydrofolate, a vital component of the folate cycle. Furthermore, the metabolites FUTP and FdUTP directly incorporate into RNA and DNA, respectively, leading to direct genetic damage and subsequent cell death. Therefore, apart from *TYMS*, the genes involved in the folate cycle and DNA repair pathways play an important role in the PD of capecitabine (Figure 1) [8]. Indeed, numerous studies have investigated the impact of several SNPs in genes related to capecitabine’s PD, such as *TYMS*, enolase superfamily member 1 gene (*ENOSF1*), methylenetetrahydrofolate reductase gene (*MTHFR*), ERCC excision repair 1, endonuclease non-catalytic subunit gene (*ERCC1*), ERCC excision repair 2, TFIIH core complex helicase subunit gene (*ERCC2*), X-ray repair cross-complementing 1 gene (*XRCC1*), and X-ray repair cross-complementing 3 gene (*XRCC3*) on the effectiveness of FP therapy in gastrointestinal neoplasms [9,10,11,12,13,14,15,16,17,18]. However, the available evidence remains limited and characterized by conflicting findings, necessitating further investigation and exploration.

Evaluating the impact of SNPs in genes associated with the mechanism of action of capecitabine on drug survival is critical for optimizing future therapeutic strategies and improving clinical outcomes. This study aims to assess the influence of SNPs in genes involved in capecitabine’s PD pathway on disease-free survival (DFS) in CRC patients.

## 2. Results

### 2.1. Patient Characteristics

Sociodemographic and clinical characteristics of the study population (*n* = 142) are presented in Table 1. Median age at diagnosis was 65.00 [57.00–73.00] years. The majority of patients were men (62.68%; 89/142), had family history of cancer (59.86%; 85/142), and optimal performance status (75.35%; 107/142). All patients had European ancestry (100%; 142). Median tumor size was 4.30 [3.00–6.00] cm. Predominantly, patients had primary tumors located in the colon (57.04%; 81/142), low histological grades (88.73%; 126/142), and advanced stages of cancer at diagnosis (88.03%; 125/142). Approximately half of the patients received capecitabine in combination regimens (54.23%; 77/142). Median duration of treatment was 5.43 [5.83–4.18] months. Patients were followed up for a median period of 13.80 [7.28–22.05] months. 

### 2.2. Genotype Frequency and Distribution

**Figure 2:**
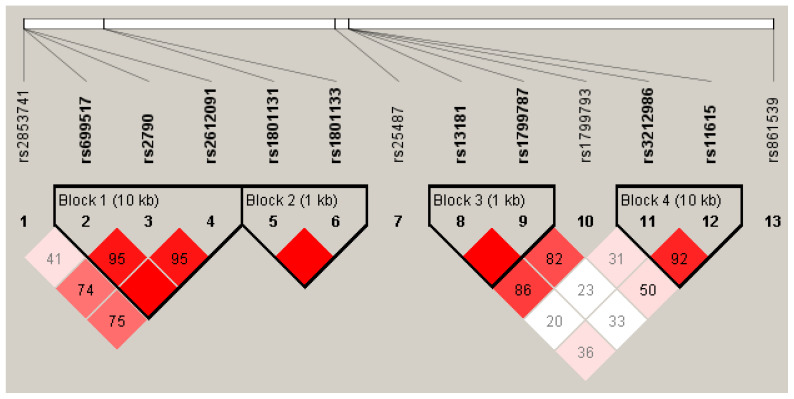
Linkage disequilibrium of selected SNPs. Color scheme: bright red: D’ = 1, LOD ≥ 2, shades of pink/red: D’ < 1, LOD ≥ 2; white: D’ < 1, LOD < 2.

Genotyping was successful for the 13 selected SNPs. Minor allele frequency (MAF) was >1% for all analyzed SNPs (Table S1). Additionally, except for *TYMS*/*ENOSF1* rs2790 (*p* = 0.033), all the SNPs examined adhered to Hardy Weinberg equilibrium (HWE) (*p* > 0.05) (Table S2). Nonetheless, no statistically significant differences were detected when comparing the observed *TYMS*/*ENOSF1* rs2790-G allele frequency with that reported for the Iberian population in Spain (IBS) at 0.274 vs. 0.248, *p* = 0.498 (Table S3). None of the MAF of the SNPs included in the study population showed significant differences with the IBS (*p* > 0.050; Table S3). Linkage disequilibrium (LD) analysis revealed that the SNPs *TYMS*/*ENOSF1* rs69957–*ENOSF1* rs2612091, *MTHFR* rs1801131–1801133, *ERCC2* rs13181–rs1799787, and *ERCC1* rs3212986–rs11615 were in strong LD (D’ > 0.900) (Figure 2; Table S4). Tables S5–S8 present the frequency estimates of haplotypes in the *TYMS*/*ENOSF1* gene region and in the genes *MTHFR*, *ERCC2*, and *ERCC1.*

### 2.3. Influence of Non-Genetic Factors on Disease-Free Survival

**Figure 3:**
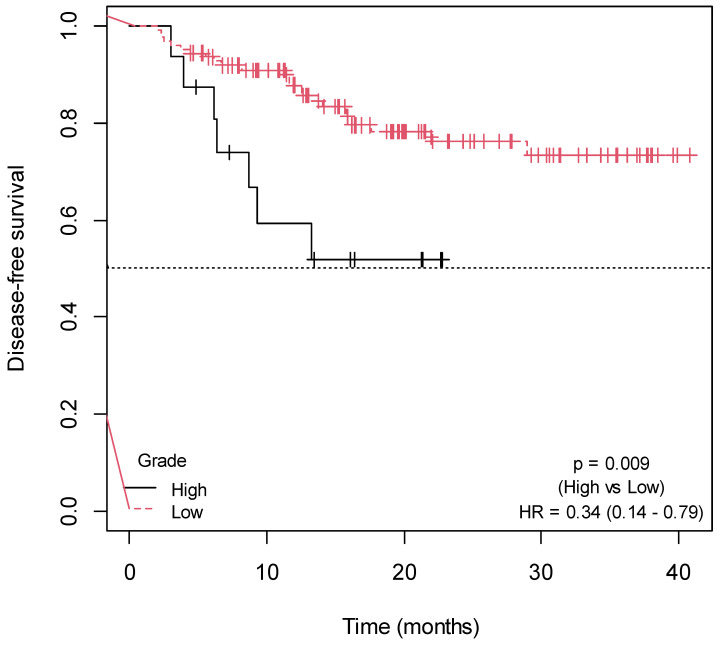
Kaplan–Meier curves for the association of DFS with histological grade.

**Figure 4:**
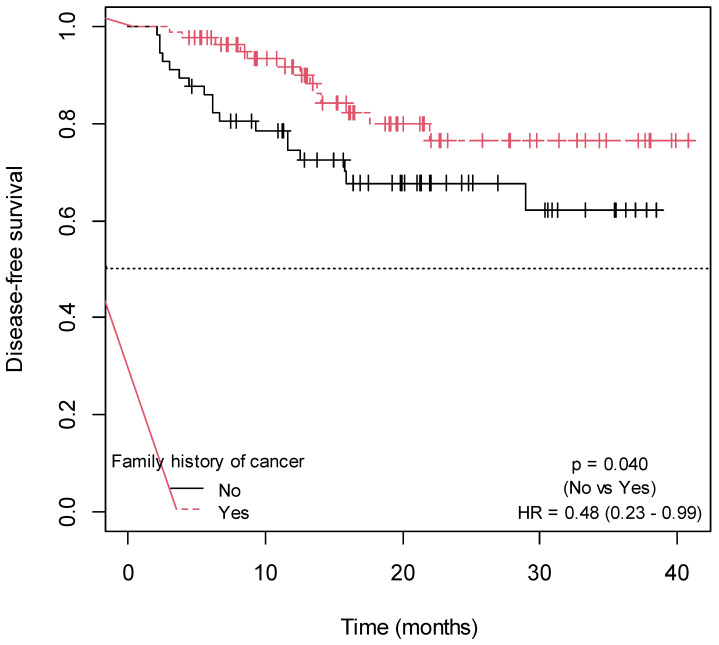
Kaplan–Meier curves for the association of DFS with family history of cancer.

Patients with low histological tumor grade (*p* = 0.009; HR = 0.34; 95% CI = 0.14–0.79; Figure 3) and a positive family history of cancer (*p* = 0.040; HR = 0.48; 95% CI = 0.23–0.99; Figure 4) exhibited a significantly higher DFS (Table S9). There was a trend towards increased DFS in patients with primary tumor located in the rectum (*p* = 0.080; HR = 0.50; 95% CI = 0.23–1.10) (Figure S1; Table S9). In contrast, a trend of association between larger tumor size and shorter DFS was observed (*p* = 0.080; HR = 1.14; 95% CI = 0.97–1.32) (Table S9). No association was found between DFS, and the rest of the sociodemographic and clinical variables collected in the study (Figures S2–S5; Table S9).

### 2.4. Influence of SNPs in Capecitabine Pharmacodynamics on Disease-Free Survival

**Figure 5:**
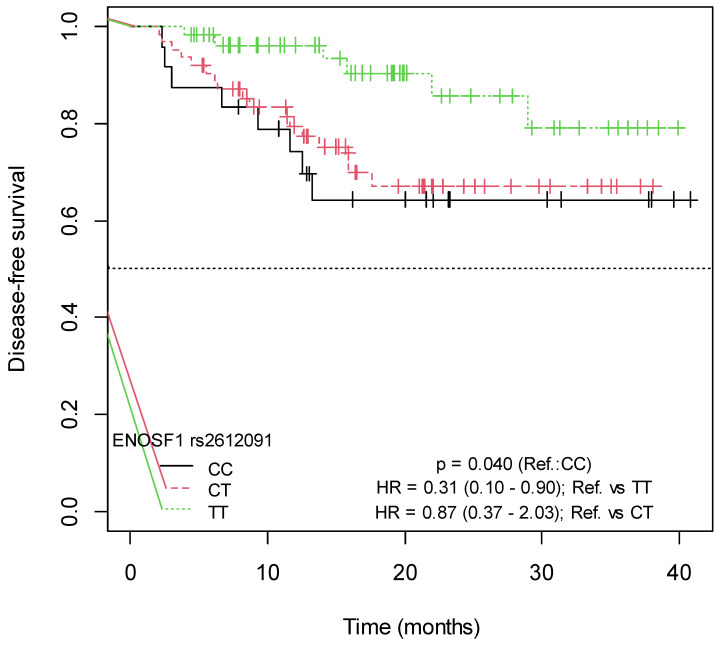
Kaplan–Meier curves for the association of DFS with *ENOSF1* rs2612091-TT (Allele C vs. genotype TT).

**Figure 6:**
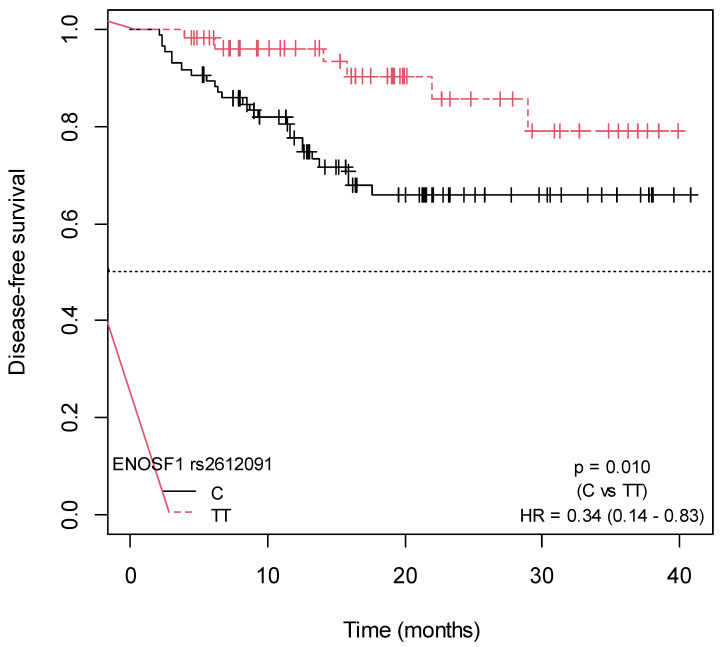
Kaplan–Meier curves for the association of DFS with *ENOSF1* rs2612091-TT (Genotype CT vs. TT).

A significant association was found between the *ENOSF1* rs2612091-TT genotype and increased DFS (*p* = 0.010; HR = 0.34; 95% CI = 0.14–0.83, for C vs. TT, and *p* = 0.040; HR = 0.31; 95% CI = 0.10–0.90, for CC vs. TT) (Table S10). The Kaplan–Meier survival curves illustrating the associations of DFS with the *ENOSF1* rs2612091 SNP are presented in Figure 5 and Figure 6. In the bivariate Cox model, an association was observed between the *MTHFR* rs1801131-T allele and decreased DFS, as all events occurred in patients carrying this allele (*p* = 0.040) (Table S10). No association was found between DFS and the remaining studied SNPs (Figures S6–S17; Table S10).

Haplotype analysis showed an association between haplotype in *TYMS*/*ENOSF1* region (rs2790, rs699517, and rs2612091) ACT and increased DFS (*p* = 0.012; HR = 0.37; 95% CI = 0.17–0.80; for ACC vs. ACT) (Table 2). No significant association was found between the remaining studied haplotypes and DFS (Tables S11–S13). 

The multivariate analysis revealed significant associations between higher DFS and positive family history of cancer (*p* = 0.017; HR = 0.41; 95% CI = 0.20–0.85), low tumor grade at diagnosis (*p* = 0.004; HR = 0.29; 95% CI = 0.12–0.68), as well as the TT genotype of the *ENOSF1* rs2612091 SNP (*p* = 0.009; HR = 0.30; 95% CI = 0.12–0.74). All associations remained significant after multiple comparison correction. The multivariate analysis of the association of DFS with non-genetic variables and the studied SNPs is presented in Table 3.

## 3. Discussion

Investigating the influence of genetic variations on treatment efficacy holds significant scientific value. The absence of validated genetic biomarkers for predicting survival in FP-based treatment strategies highlights the importance of further research in this area. Our study investigated the role of SNPs related to capecitabine PD in treatment survival. Our findings revealed that the *ENOSF1* rs2612091-TT genotype and the *TYMS*/*ENOSF1* ACT haplotype were associated with longer DFS in CRC patients undergoing adjuvant capecitabine-based therapy. Furthermore, family history of cancer and low histological grade were identified as potential factors contributing to improved DFS. Understanding the role of these genetic factors could bear significant implications for personalized medicine, enabling clinicians to tailor capecitabine treatments based on individual genetic profiles and potentially leading to improved treatment survival rates.

A positive family history of cancer is a well-known risk factor for developing the disease, but its influence on prognosis remains uncertain [19]. Our results suggest a significant association between positive family history of cancer and improved DFS. These findings are in line with previous studies conducted in patient cohorts with colon, gastric, and prostate cancer [19,20,21]. Patients with family history of cancer tend to demonstrate (a) heightened awareness and vigilance regarding the condition, which leads to early detection and initiation of treatment, potentially improving survival outcomes; and (b) proactive behavior in maintaining a healthy lifestyle and actively participating in treatment. This behavior contributes to enhanced disease management and treatment adherence, consequently yielding improved response and prognosis [19,20,21].

Histological grading is a routinely used prognostic marker in CRC [22,23]. Specifically, a high grade is recognized as a negative prognostic factor [22,24]. This statement correlates with what was found in our study, where a low histological grade was associated with better survival outcomes among the CRC patients investigated. Existing evidence indicates that low-grade tumors exhibit less aggressive and invasive behavior than high-grade tumors and improved survival outcomes [22,25,26,27,28,29,30]. However, it is important to mention that other factors, such as tumor stage, patient age, and general health status, have also been recognized as factors that may influence disease prognosis [23,24,31].

The *ENOSF1* gene, responsible for encoding the protein mitochondrial enolase superfamily member 1 (ENOF1), exhibits three distinct isoforms. One of these isoforms has been identified as an l-fuconate dehydratase, actively participating in the catabolism of L-fucose, a sugar integral to the carbohydrate composition of cellular glycoproteins. ENOF1 catalyzes the dehydration of L-fuconate to 2-keto-3-deoxy-L-fuconate by extracting the 2-proton, generating an enediolate intermediate stabilized by the presence of magnesium ion [32,33]. Although the role of ENOF1 in the mechanism of action of FP is not fully understood, the *ENOSF1* gene has been extensively studied for its influence on the toxicity and effectiveness of FP-based treatments [18,32,34,35]. The rationale for investigating this gene is based on its location in the genome and its potential effects on proteins that are formally related to the PD of FP. The *ENOSF1* gene partially overlaps with the *TYMS* gene (in the *TYMS*/*ENOSF1* region) on chromosome 18 and is transcribed in the opposite direction to the latter [36]. It has been suggested that *ENOSF1* is involved in the regulation of the expression of *TYMS*, thus affecting the outcomes of FP-based therapy [37]. Moreover, the ENOF1 enzyme has been indicated to be more sensitive to the cytotoxic effects of FP than TS [38]. In-depth analysis of protein–protein interactions through the STRING interactomics platform reveals significant insights into the relationship between TS and ENOF1. Despite ENOF1 appearing to interact with approximately 10 proteins (TS, CLUL1, NPL, and DPH5, among others), it exhibits a high-confidence association score (>0.7) solely with TS (0.978). This substantial score is primarily attributed to text mining (0.957), presenting a low score in the co-expression type of association (0.075). Hence, TS emerges as the sole protein involved in this context, although not through direct physical interaction. This observation underscores the need for further exploration in understanding the functional details underlying *ENOSF1* [39]. The *ENOSF1* rs2612091 variant (g.683607C>T), located in the intronic region of the gene, has been associated with *ENOSF1* mRNA expression and FP-therapy outcomes [18,32,34,35]. In our study, we observed that patients carrying genotype *ENOSF1* rs2612091-TT exhibited superior DFS compared to C allele carriers. These findings align with the results reported by Meulendijks et al. (Caucasian population; The Netherlands; n = 185) in a pooled analysis of three prospective studies that involved gastric cancer patients undergoing capecitabine-based regimens. They observed a significant association between the *ENOSF1* rs2612091-CC genotype and lower overall survival (OS) (*p* = 0.041; HR = 1.50; 95% CI = 1.00–2.30, for TT/CT vs. CC) after adjusting for clinical covariates and the *TYMS* variable number tandem repeats (VNTR) variant (in moderate LD with *ENOSF1* rs2612091). Although, it is important to mention that the VNTR variant exhibited only a mild trend toward an association with OS (*p* = 0.076). Subgroup analysis based on disease extension revealed that the impact of *ENOSF1* rs2612091 was more pronounced in patients with locally advanced disease. In this context, patients carrying the TT genotype demonstrated notably enhanced OS (*p* = 0.001; HR = 6.5; 95% CI = 2.10–20.00, for CC vs. TT or CT) as well as progression-free survival (PFS) (*p* = 0.005; HR = 4.3, for CC vs. TT or CT) [35]. However, our findings contrasted with those reported by Arjmandi et al. (Middle Eastern population; Iran; n = 97) in a study involving gastric cancer patients treated with neoadjuvant 5-FU-based therapy. The researchers reported that the *ENOSF1* rs2612091-CC genotype was linked to an increased response to 5-FU compared to the T allele (*p* = 0.017) [18]. These contradictory findings may be explained by differences in the ethnicities of the populations studied, the type of therapeutic regimen under investigation, and the sample sizes of the studies. As there are no further studies exploring the impact of the *ENOSF1* rs2612091 SNP, the disparities in these reported findings underscore the need to assess this discovery within larger prospective cohorts to establish its authentic association.

While our study has provided valuable insights into the influence of SNPs in capecitabine’s PD on therapy effectiveness and has revealed the potential relevance of the *TYMS*/*ENOSF1* genetic region on DFS, it is important to recognize specific limitations that might affect the interpretation of the results. Firstly, the relatively small sample size in our study may limit the ability to detect significant differences in DFS. Secondly, the ambispective nature of the study could lead to differences in follow-up duration between retrospective and prospective cohorts, potentially influencing the estimation of survival rates. Thirdly, the exclusive inclusion of patients with European ancestry may impact the generalizability of our findings. Lastly, the median follow-up time might be considered relatively short, possibly leading to the underrepresentation of long-term events. 

In summary, the findings of our study reveal a significant association between *ENOSF1* rs2612091-CC genotype ACT haplotype in the *TYMS*/*ENOSF1* gene region with DFS in CRC patients under capecitabine-based adjuvant therapy, suggesting their potential as promising genetic biomarkers of treatment prognosis. However, further research with prospective designs, longer follow-up times to consider their impact on OS, and larger sample sizes are necessary to validate and strengthen these results, ensuring their clinical applicability.

## 4. Materials and Methods

### 4.1. Design and Settings

The research was designed as an observational ambispective study. DNA samples from CRC patients treated at the University Hospital Virgen de las Nieves, Granada, Spain, who had been previously genotyped for *DPYD* variants, were requested to the Andalusian Public Health System biobank and stored at −40 °C. The study was conducted according to the principles of the Declaration of Helsinki and approved by the Biomedical Research Ethics Committee of Granada (Identification code 0632-M2-20, July 2020). The DNA samples included had written informed consent for donation to the Biobank of the Andalusian Public Health System and were identified by alphanumeric codes.

### 4.2. Study Population

**Figure 7:**
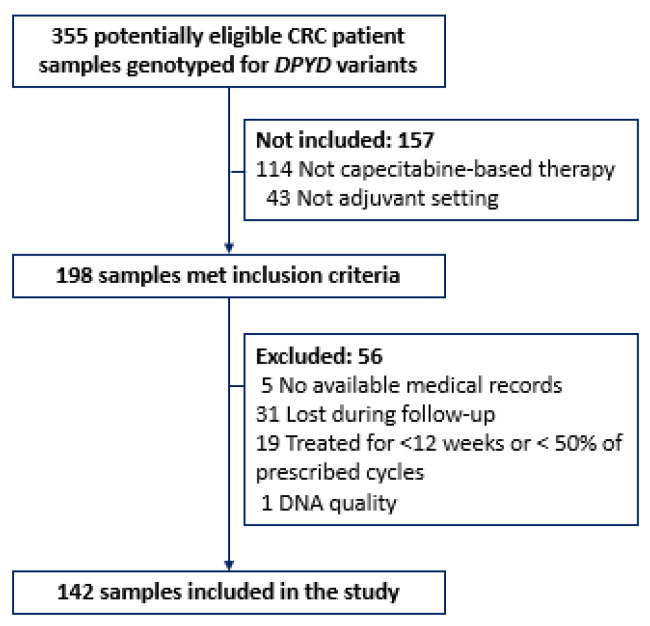
Patient flow diagram.

The DNA samples of CRC patients were included in the study when meeting eligibility criteria. The inclusion criteria encompassed patients with advanced CRC who were candidates for capecitabine-based adjuvant therapy, aged ≥18 years, and possessed a performance status ≤ 2. Patients with previous malignancies, without available medical records, missing data, lost during follow-up, with abnormal liver or renal function, treated for less than 12 weeks or less than half of the prescribed cycles, and DNA samples with low quality were excluded from the study. The number of CRC patients with DNA samples available during the study recruitment period and who met selection criteria determined the sample size. From 2019 to 2022, 355 CRC patients underwent genotyping for *DPYD* variants. Among them, 198 individuals met the inclusion criteria, while 56 were eliminated due to exclusion criteria, resulting in a final cohort of 142 patients included in the study (Figure 7).

Capecitabine (750–2150 mg, twice daily) was administered for 14 days in 3-week cycles, either as monotherapy or in combination with other antineoplastic strategies, such as radiotherapy, oxaliplatin, or immunotherapy. Patient follow-up was performed by the medical oncology department before each new treatment cycle, every 3–6 months after treatment completion in the first year, and once a year thereafter. Follow-up visits were documented in patients’ medical records. Treatment continued until completion of scheduled cycles, disease progression, death, or occurrence of unacceptable toxicity. 

### 4.3. Variables

#### 4.3.1. Endpoints

The primary endpoint of this study was DFS, defined as the time from the initiation of adjuvant therapy to disease relapse or death from any cause. Patients who were alive and without relapse at the end of follow-up were censored. Survival data were obtained from the medical records during each follow-up examination.

#### 4.3.2. Sociodemographic and Clinical Data

Sociodemographic and clinical characteristics of the patients included, such as age at CRC diagnosis, sex, primary tumor site, cancer stage (categorized as 0-IIC/IIIA-IV), histological grade (categorized as high/low), performance status, family history of cancer, and type of capecitabine-based adjuvant regimen (categorized as monotherapy/combination), were obtained from medical records.

#### 4.3.3. Genotyping Data

Samples containing 50 uL DNA were quantified using a NanoDrop 2000 UV spectrophotometer (Thermo Fisher Scientific, Waltham, MA, USA). A total of 13 SNPs in 7 genes involved in capecitabine PD were selected based on their prevalence in the European population and previous research in the scientific literature [9,10,11,12,13,14,15,16,17,18,40] (Table 4). 

Genotyping was performed in duplicate on the QuantStudio 3 Real Time PCR System using TaqMan probes (Thermo Fisher Scientific, Waltham, MA, USA), according to the manufacturer protocol, in the pharmacogenetics unit laboratory of HUVN. The reactions were arranged in a 96-well plate (0.2 mL), where each well comprised the DNA sample, TaqMan genotyping master mix, and the relevant TaqMan assay designed for the targeted SNP. Negative controls and known genotypic controls were included in each PCR run. The procedure commenced with a pre-read step at 60 °C for 30 s. Following this, an initial denaturation and enzyme activation step was performed at 95 °C for 10 min. The subsequent denaturation phase occurred at 95 °C for 15 s, repeated across 40 cycles. This denaturation step was followed by an annealing and extension phase at 60 °C for 60 s during each cycle. The post-read step concluded the cycle at 60 °C for 30 s. Real-time PCR data were analyzed using the QuantStudio 3/5 Real-Time PCR Software v.1.5.1. The genotyping results were interpreted based on the fluorescence signals and allelic discrimination plots generated during the amplification process. Call rates were >98%. Additionally, 10% of the samples were analyzed in duplicate with a concordance of 100%.

### 4.4. Statistical Analysis

Normality was verified by Kolmogorov–Smirnov test. Quantitative data were tabulated as median [p25–p75] and qualitative data were expressed as frequencies and percentages. Genotype frequencies of all the selected SNPs were assessed for HWE. MAF comparisons between the study population and the IBS population were conducted using the chi-square test to assess for significant differences. PLINK v1.9 and Haploview v.4.1 were used for the analysis and visualization of LD plots [41,42]. THESIAS software v.3.1 was used for haplotype frequency estimation and association analysis between haplotypes and DFS [43]. In bivariate analysis, Kaplan–Meier survival curves were generated to estimate and compare median DFS across sociodemographic, clinical, and SNP variables. Survival distributions were compared with log-rank test and univariate Cox proportional hazards regression model. Multivariate Cox hazards regression model including variables significantly associated with DFS in bivariate analysis was performed using a backward stepwise selection method. Statistical significance was established with a *p*-value < 0.05. False discovery rate (FDR) correction was applied to adjust *p*-values for multiple comparisons. An FDR threshold of 5% was set. Statistical analyses were conducted using PLINK v1.9 and R Software v.4.2.2 (R Foundation for Statistical Computing, Vienna, Austria) [41,44].

## Figures and Tables

**Table 1:** Sociodemographic and clinical characteristics of 142 CRC patients.

Characteristic	*n* (%)	Median [p25–p75]
Sex		
Female	53 (37.32)	-
Male	89 (62.68)	-
Age at CRC diagnosis (years)	-	65.00 [57.00–73.00]
Family history of cancer		
Yes	85 (59.86)	-
No	57 (40.14)	-
Performance status		
0	107 (75.35)	-
1	28 (19.72)	-
2	7 (4.93)	-
Primary tumor site		
Colon	81 (57.04)	-
Rectum	61 (42.96)	-
Primary tumor size (cm)	-	4.30 [3.00–6.00]
Stage		
0-IIC	17 (11.97)	-
IIIA-IV	125 (88.03)	-
Grade		
Low	126 (88.73)	-
High	16 (11.27)	-
Capecitabine-based regimen		
Monotherapy	65 (45.77)	-
Combination	77 (54.23)	-

CRC: colorectal cancer.

**Table 2:** Effect estimation of haplotypes in *TYMS*/*ENOSF1* gene region on disease-free survival.

H	*TYMS*/*ENOSF1* rs2790	*TYMS*/*ENOSF1* rs699517	*ENOSF1* *rs2612091*	Frequency	HR (95% CI)	*p*-Value
0	A	C	C	0.3830	1.00	-
1	G	T	T	0.2670	0.58 (0.31–1.07)	0.083
2	A	C	T	0.2150	0.37 (0.17–0.80)	0.012
3	A	T	T	0.1270	0.89 (0.36–2.15)	0.796
4	G	C	C	0.0040	-	-
5	G	C	T	0.0040	-	-
Goodness-of-fit: −2 × Log-likelihood (with covariates) = 278.098421						
Goodness-of-fit: −2 × Log-likelihood (without covariates) = 285.795061						
df = 3; model *p*-value: 0.052						

df: degrees of freedom; H: haplotype; HR: hazard ratio; 95% CI: 95% confidence interval.

**Table 3:** Multivariate analysis of disease-free survival associations with sociodemographic, clinical, and genetic variables.

*ENOSF1* rs2612091 (TT) Family history of cancer (Yes) Grade (low)	**Disease-Free Survival**		
	HR (95% CI)	*p*-value	*p*-BH *
	0.30 (0.12–0.74) 0.41 (0.20–0.85) 0.29 (0.12–0.68)	0.009 0.017 0.004	0.013 0.017 0.012
Model *p*-value < 0.001			

HR: hazard ratio, 95% CI: 95% confidence interval. * Benjamini–Hochberg adjusted *p*-value.

**Table 4:** Selected SNPs in capecitabine pharmacodynamic pathway.

Gene	Location *	SNP rs	SNP Type	TaqMan Assay ID
*TYMS*	Chr. 18, g.657352T>C	rs2853741	2KB upstream variant	C__26612342_10
*TYMS-ENOSF1*	Chr. 18, g.673086A>G	rs2790	3 primer UTR variant/ Intron variant	C___7486263_10
*TYMS-ENOSF1*	Chr. 18, g.673016C>T	rs699517	3 primer UTR variant/ Non-coding transcript variant	C___7486269_10
*ENOSF1*	Chr. 18, g.683607C>T	rs2612091	Intron variant	C__15908768_10
*MTHFR*	Chr. 1, g.11794419T>G	rs1801131	Missense variant (p.Glu469Ala)	C____850486_20
*MTHFR*	Chr. 1, g.11796321G>A	rs1801133	Missense variant (p.Ala262Val)	C_1202883_20
*ERCC1*	Chr. 19, g.45420395A>G	rs11615	Synonymous variant (p.Asn118=)	C__2532959_10
*ERCC1*	Chr. 19, g.45409478C>A	rs3212986	3 primer UTR variant	C__2532948_10
*ERCC2*	Chr. 19, g.45352886G>A	rs1799787	Intron variant	C__11973901_10
*ERCC2*	Chr. 19, g.45351661T>G	rs13181	Missense variant (p.Lys751Gln)	C__3145033_10
*ERCC2*	Chr. 19, g.45364001C>T	rs1799793	Missense variant (p.Asp312Asn)	C__3145050_10
*XRCC1*	Chr. 19, g.43551574T>C	rs25487	Missense variant (p.Gln399Arg)	C__622564_10
*XRCC3*	Chr. 14, g.103699416G>A	rs861539	Missense variant (p.Thr241Met)	C__8901525_10

* Placement: GRCh38.p14. *ERCC1*: ERCC excision repair cross-complementing group 1; *ERCC2*: ERCC excision repair cross-complementing group 2; *ENOSF1*: enolase superfamily member 1; *MTHFR*: methylenetetrahydrofolate reductase; SNP: single-nucleotide polymorphism; *TYMS*: thymidylate synthetase; *XRCC1*: X-ray repair cross-complementing 1; *XRCC3*: X-ray repair cross-complementing 3.

## Data Availability

Data are unavailable due to privacy and ethical restrictions.

## Supplementary Materials

The supporting information can be downloaded at: https://www.mdpi.com/article/10.3390/ijms25010104/s1.

## Abbreviations

5-FU: 5-fluorouracil; 5,10-MTHF: 5,10-methylenetetrahydrofolate; ADC: Adenocarcinomas; CRC: colorectal cancer; DFS: disease-free survival; *DPYD*: dihydropyrimidine dehydrogenase gene; *ERCC1*: ERCC excision repair 1, endonuclease non-catalytic subunit; *ERCC2*: ERCC excision repair 2, TFIIH core complex helicase subunit; ENOF1: mithocondrial-enolase-superfamily-member-1 protein; *ENOSF1*: enolase-superfamily-member-1 gene; FDR: false discovery rate; FdUMP: fluorodeoxyuridine monophosphate; FdUTP: fluorodeoxyuridine triphosphate; FP: fluoropyrimidines; FUTP: fluorouridine triphosphate; HR: hazard ratio; HWE: Hardy–Weinberg equilibrium; LD: linkage disequilibrium; MAF: minor allele frequency; *MTHFR*: methylenetetrahydrofolate-reductase; OS: overall survival; PD: pharmacodynamics; PFS: progression-free survival; SNP: single nucleotide polymorphism; TS: thymidylate synthase protein; *TYMS*: thymidylate-synthase gene; VNTR: variable number tandem repeats; *XRCC1*: X-ray repair cross-complementing 1; *XRCC3*: X-ray repair cross-complementing 3.
