# Supplementary material for: Role of Single-Nucleotide Polymorphisms in Genes Implicated in Capecitabine Pharmacodynamics on the Effectiveness of Adjuvant Therapy in Colorectal Cancer
Source: Int J Mol Sci. 2023 Dec 20;25(1):104. doi: 10.3390/ijms25010104 (PMC10778960; doi:10.3390/ijms25010104)
Supplement: Supplementary file 1 [file ijms-25-00104-s001.zip › ijms-2715263-supplementary-modified.pdf]

Supplementary material:

Role of Single Nucleotide Polymorphisms in Genes implicated in Capecitabine Pharmacodynamics on the Effectiveness of Adjuvant Therapy in Colorectal Cancer

Table S1. SNPs minor allele frequency.

Chr	SNP	Minor Allele	Major Allele	MAF
1	rs1801131	G	T	0.271
1	rs1801133	A	G	0.426
14	rs861539	A	G	0.404
18	rs2853741	T	C	0.345
18	rs2790	G	A	0.274
18	rs699517	T	C	0.394
18	rs2612091	C	T	0.387
19	rs11615	G	A	0.387
19	rs3212986	A	C	0.250
19	rs1799787	A	G	0.278
19	rs13181	G	T	0.341
19	rs1799793	T	C	0.295
19	rs25487	T	C	0.366

Chr: Chromosome; MAF: Minor allele frequency.

Table S2. Hardy-Weinberg Equilibrium.

Chr	SNP	Minor Allele	Major Allele	Genotype counts	Observed heterozygosity	Expected heterozygosity	p-value
1	rs1801131	G	T	9/59/74	0.4155	0.3952	0.6722
1	rs1801133	A	G	26/69/47	0.4859	0.4891	1
14	rs861539	A	G	26/63/53	0.4437	0.4819	0.3837
18	rs2853741	T	C	19/60/63	0.4225	0.452	0.4588
18	rs2790	G	A	16/46/80	0.3239	0.3984	0.0336
18	rs699517	T	C	26/60/56	0.4225	0.4777	0.1641
18	rs2612091	C	T	24/62/56	0.4366	0.4746	0.3765
19	rs11615	G	A	18/74/50	0.5211	0.4746	0.291
19	rs3212986	A	C	8/55/79	0.3873	0.375	0.8245
19	rs1799787	A	G	9/61/72	0.4296	0.4016	0.531
19	rs13181	G	T	16/65/61	0.4577	0.4498	1
19	rs1799793	T	C	12/60/70	0.4225	0.4166	1
19	rs25487	T	C	17/70/55	0.493	0.4642	0.5872

Chr: chromosome; SNP: single nucleoWide polymorphism.

Table S3. Comparison of Minor Allele Frequency between Study Cohort and Spanish Population.

Gene	SNP rs	Minor allele	Study Cohort MAF % (count)	IBS population MAF % (count)	p-value*
MTHFR	rs1801131	G	0.271 (77)	0.271 (58)	0.998
MTHFR	rs1801133	A	0.426 (121)	0.444 (95)	0.767
XRCC3	rs861539	A	0.404 (115)	0.397 (85)	0.861
TYMS	rs2853741	T	0.345 (98)	0.383 (82)	0.380
TYMS-ENOSF1	rs2790	G	0.274 (78)	0.248 (53)	0.498
TYMS-ENOSF1	rs699517	T	0.394 (112)	0.350 (75)	0.316
ENOSF1	rs2612091	C	0.387 (110)	0.463 (99)	0.091
ERCC1	rs11615	G	0.387 (110)	0.369 (79)	0.679
ERCC1	rs3212986	A	0.250 (71)	0.234 (50)	0.625
ERCC2	rs1799787	A	0.278 (79)	0.280 (60)	0.956
ERCC2	rs13181	G	0.341 (97)	0.313 (67)	0.503
ERCC2	rs1799793	T	0.295 (84)	0.341 (73)	0.317
XRCC1	rs25487	T	0.366 (104)	0.421 (90)	0.218

MAF frequencies compared to IBS (data extracted from 1,000 Genomes Project Phase 3, available at: https://www.ensembl.org/index.html). MAF: minor allele frequency. IBS: Iberian populations in Spain.
* Estimated with chi-square test.

Table S4. Linkage disequilibrium.

Chr	BP	SNP	Chr	BP	SNP	R^2	D
1	11794419	rs1801131	1	11796321	rs1801133	0.380	1.000
18	657352	rs2853741	18	673086	rs2790	0.424	0.718
18	657352	rs2853741	18	683607	rs2612091	0.204	0.749
18	673086	rs2790	18	673016	rs699517	0.542	0.953
18	673086	rs2790	18	683607	rs2612091	0.228	0.954
18	673016	rs699517	18	683607	rs2612091	0.449	1.000
19	45420395	rs11615	19	45409478	rs3212986	0.437	0.918
19	45352886	rs1799787	19	45351661	rs13181	0.752	1.000
19	45352886	rs1799787	19	45364001	rs1799793	0.633	0.829
19	45351661	rs13181	19	45364001	rs1799793	0.619	0.883

BP: Base-pair physical position ; Chr: chromosome; SNP: single nucleotide polymorphism.

Table S5. Haplotype frequency estimation in *TYMS/ENOSF1* gene region under linkage disequilibrium.

H	TYMS/ENOSF1 rs2790	TYMS/ENOSF1 rs699517	*ENOSF1* rs2612091	Total	Censored	Uncensored	Cumulative frequency
0	A	C	C	0.3830	0.3420	0.5320	0.383
1	G	T	T	0.2670	0.2830	0.2090	0.650
2	A	C	T	0.2150	0.2430	0.1130	0.865
3	A	T	T	0.1270	0.1220	0.1460	0.992
4	G	C	C	0.0040	0.0050	0.0010	0.996
5	G	C	T	0.0040	0.0050	0.0002	1

H: haplotype.

Table S6. Haplotype frequency estimation in gene *MTHFR* under linkage disequilibrium.

H	*MTHFR* rs1801131	*MTHFR* rs1801133	Total	Censored	Uncensored	Cumulative frequency
0	T	A	0.426	0.423	0.435	0.426
1	T	G	0.303	0.302	0.306	0.729
2	G	G	0.271	0.275	0.258	1

H: haplotype.

Table S7. Haplotype frequency estimation in gene *ERCC2* gene under linkage disequilibrium.

H	*ERCC2* rs179787	*ERCC2* rs13181	Total	Censored	Uncensored	Cumulative frequency
0	G	T	0.659	0.644	0.710	0.659
1	A	G	0.278	0.293	0.226	0.937
2	G	G	0.063	0.063	0.064	1

H: haplotype.

Table S8. Haplotype frequency estimation in gene *ERCC1* gene under linkage disequilibrium.

H	*ERCC1* rs3212986	*ERCC1* rs11615	Total	Censored	Uncensored	Cumulative frequency
0	C	A	0.600	0.592	0.629	0.600
2	A	G	0.238	0.245	0.209	0.838
1	C	G	0.150	0.146	0.161	0.988
3	A	A	0.012	0.016	0.000	1

H: haplotype.

Table S9. Association of sociodemographic and clinical characteristics with disease-free survival.

<table>
<tr><th colspan="2" rowspan="3">Characteristic</th><th colspan="9">Disease-free survival</th></tr>
<tr><th rowspan="2">n</th><th rowspan="2">Events</th><th rowspan="2">MST (m)</th><th rowspan="2">95% CI</th><th rowspan="2">p-value [a]</th><th rowspan="2">Ref. cat.</th><th colspan="3">Bivariate Cox model</th></tr>
<tr><th>HR</th><th>95% CI</th><th>p-value</th></tr>
<tr><td rowspan="2">Sex</td><td>Female</td><td>53</td><td>12</td><td>NA</td><td>29 - NA</td><td rowspan="2">0.800</td><td rowspan="2">Male</td><td rowspan="2">1.12</td><td rowspan="2">0.54 - 2.31</td><td rowspan="2">0.800</td></tr>
<tr><td>Male</td><td>89</td><td>19</td><td>NA</td><td>NA - NA</td></tr>
<tr><td rowspan="2">Family history of cancer</td><td>Yes</td><td>85</td><td>13</td><td>NA</td><td>NA - NA</td><td rowspan="2">0.040</td><td rowspan="2">No</td><td rowspan="2">0.48</td><td rowspan="2">0.23 - 0.99</td><td rowspan="2">0.050</td></tr>
<tr><td>No</td><td>57</td><td>18</td><td>NA</td><td>29.00 - NA</td></tr>
<tr><td rowspan="2">Primary tumor location</td><td>Colon</td><td>81</td><td>22</td><td>NA</td><td>29 - NA</td><td rowspan="2">0.080</td><td rowspan="2">Colon</td><td rowspan="2">0.51</td><td rowspan="2">0.23 - 1.10</td><td rowspan="2">0.080</td></tr>
<tr><td>Rectum</td><td>61</td><td>9</td><td>NA</td><td>NA - NA</td></tr>
<tr><td rowspan="2">Stage at diagnosis</td><td>0-IIC</td><td>17</td><td>3</td><td>NA</td><td>NA - NA</td><td rowspan="2">0.500</td><td rowspan="2">0-IIC</td><td rowspan="2">1.47</td><td rowspan="2">0.44 - 4.87</td><td rowspan="2">0.500</td></tr>
<tr><td>IIIA-IV</td><td>125</td><td>28</td><td>NA</td><td>NA - NA</td></tr>
<tr><td rowspan="2">Histological grade</td><td>High</td><td>16</td><td>7</td><td>NA</td><td>8.67 - NA</td><td rowspan="2">0.009</td><td rowspan="2">Alto</td><td rowspan="2">0.34</td><td rowspan="2">0.14 - 0.79</td><td rowspan="2">0.030</td></tr>
<tr><td>Low</td><td>126</td><td>24</td><td>NA</td><td>NA - NA</td></tr>
<tr><td rowspan="3">PS ECOG</td><td>0</td><td>107</td><td>23</td><td>NA</td><td>NA - NA</td><td rowspan="3">0.700</td><td rowspan="3">0</td><td>1.00</td><td>-</td><td rowspan="3">0.700</td></tr>
<tr><td>1</td><td>28</td><td>7</td><td>NA</td><td>29.00 - NA</td><td>1.31</td><td>0.56 - 3.06</td></tr>
<tr><td>2</td><td>7</td><td>1</td><td>NA</td><td>NA - NA</td><td>0.65</td><td>0.08 - 4.88</td></tr>
<tr><td rowspan="2">Type of adjuvant treatment</td><td>Capecitabine monotherapy</td><td>65</td><td>13</td><td>NA</td><td>NA - NA</td><td rowspan="2">0.700</td><td rowspan="2">mono-therapy</td><td rowspan="2">1.14</td><td rowspan="2">0.56 - 2.33</td><td rowspan="2">0.700</td></tr>
<tr><td>Capecitabine combination</td><td>77</td><td>18</td><td>NA</td><td>NA - NA</td></tr>
<tr><td colspan="2">Age at diagnosis</td><td>142</td><td>31</td><td>-</td><td>-</td><td>0.400</td><td>-</td><td>1.01</td><td>0.97 - 1.05</td><td>0.400</td></tr>
<tr><td colspan="2">Primary tumor size</td><td>142</td><td>31</td><td>-</td><td>-</td><td>0.080</td><td>-</td><td>1.14</td><td>0.97 - 1.32</td><td>0.100</td></tr>
</table>

CRC, colorectal cancer; m: months; MST: median survival time; n: number of patients; NA: not available. HR: Hazard ratio, 95% CI: 95% confidence interval; Ref. cat.: reference category.

a. Log-rank p-value.

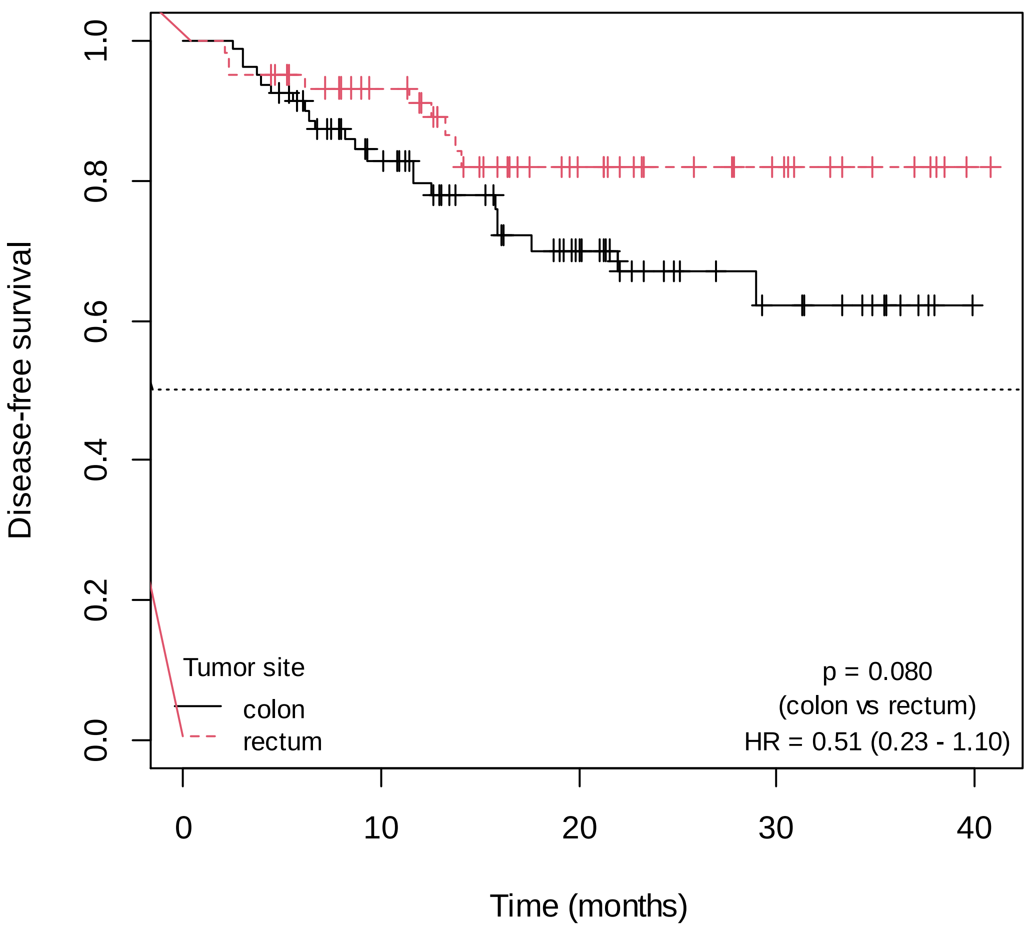

Figure S1. Kaplan-Meier survival curve for the association of DFS with primary tumor location

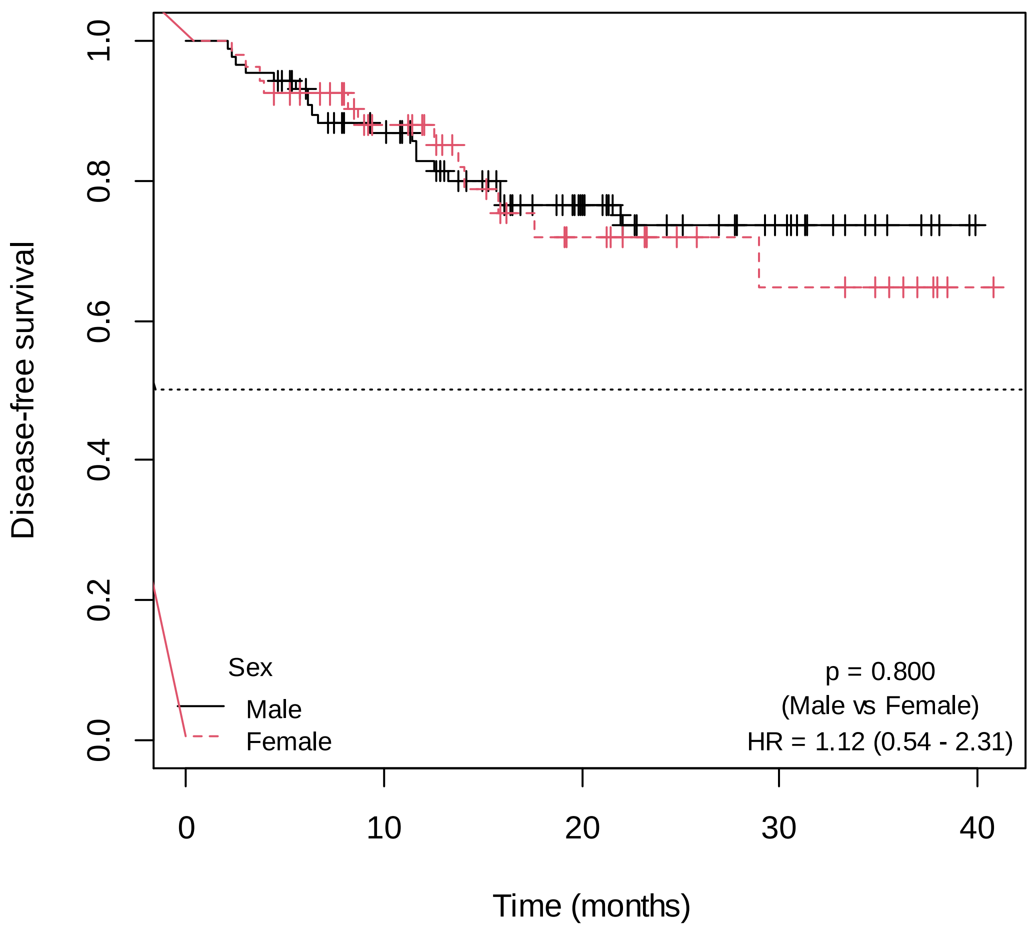

Figure S2. Kaplan-Meier survival curve for the association of DFS with sex

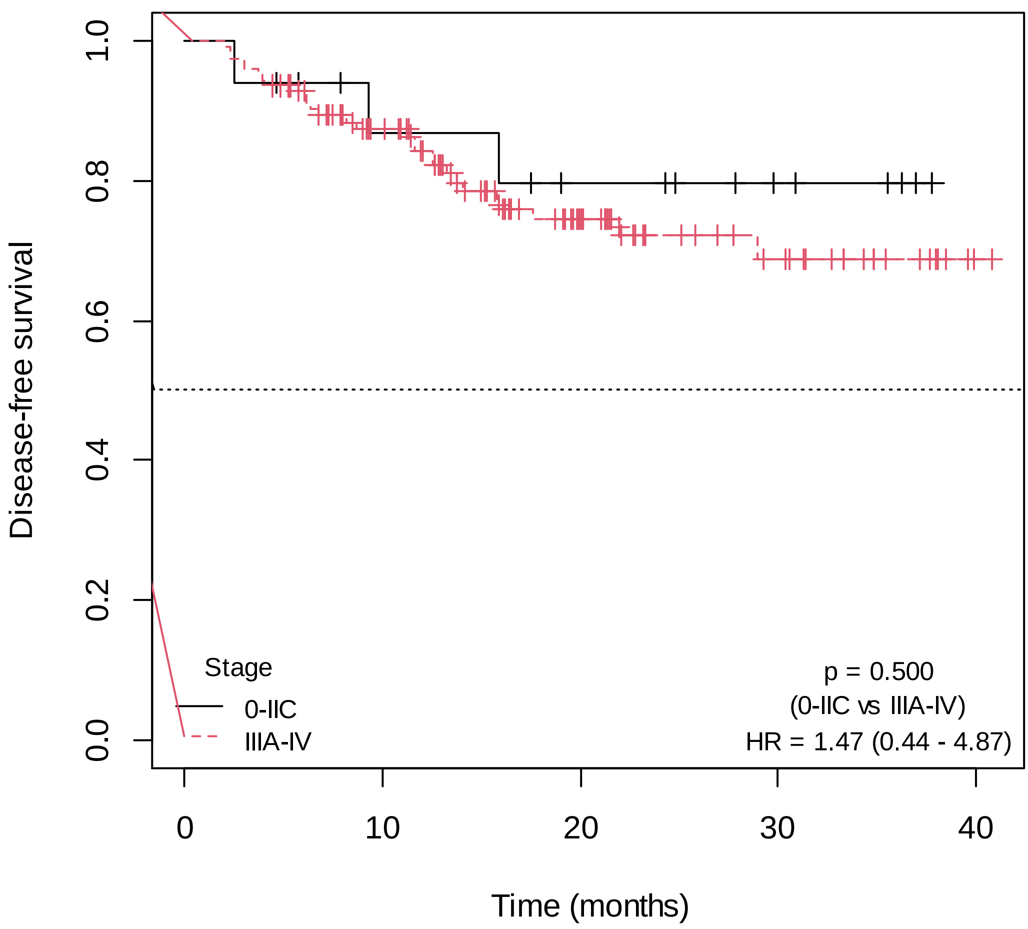

Figure S3. Kaplan-Meier survival curve for the association of DFS with stage at diagnosis

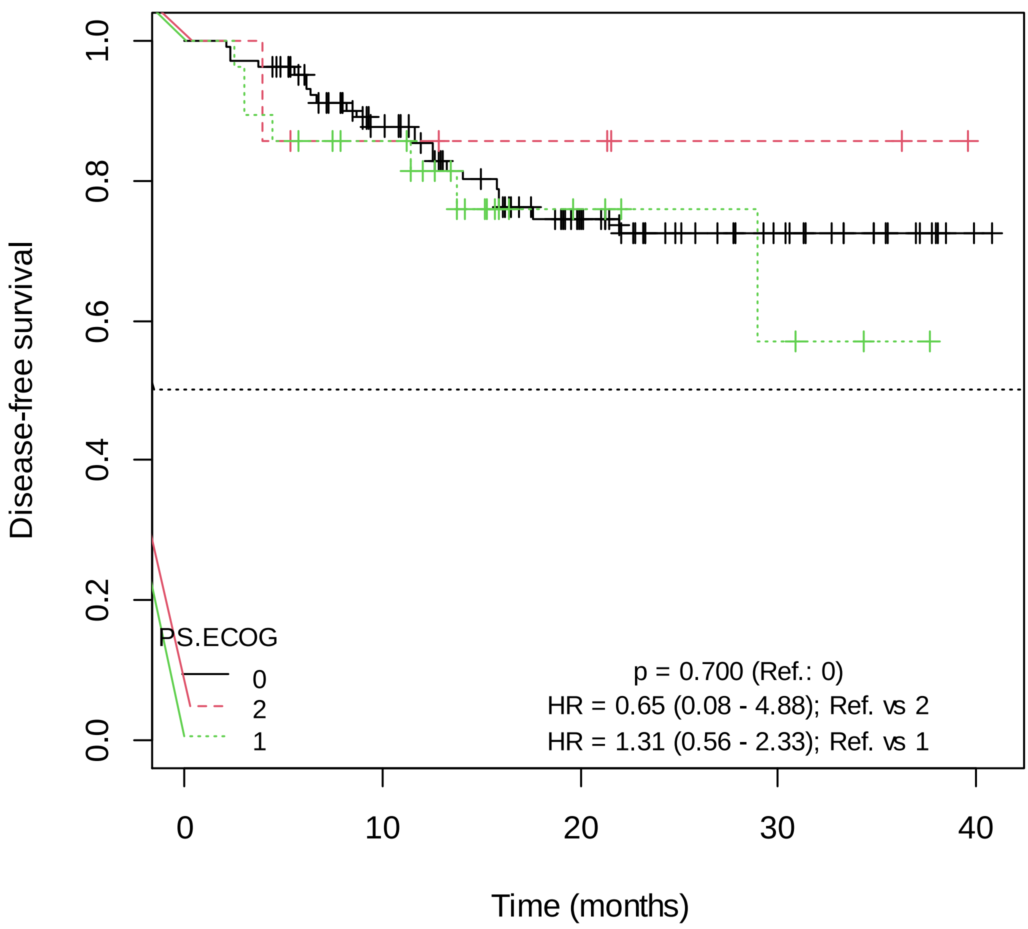

Figure S4. Kaplan-Meier survival curve for the association of DFS with PS. ECOG

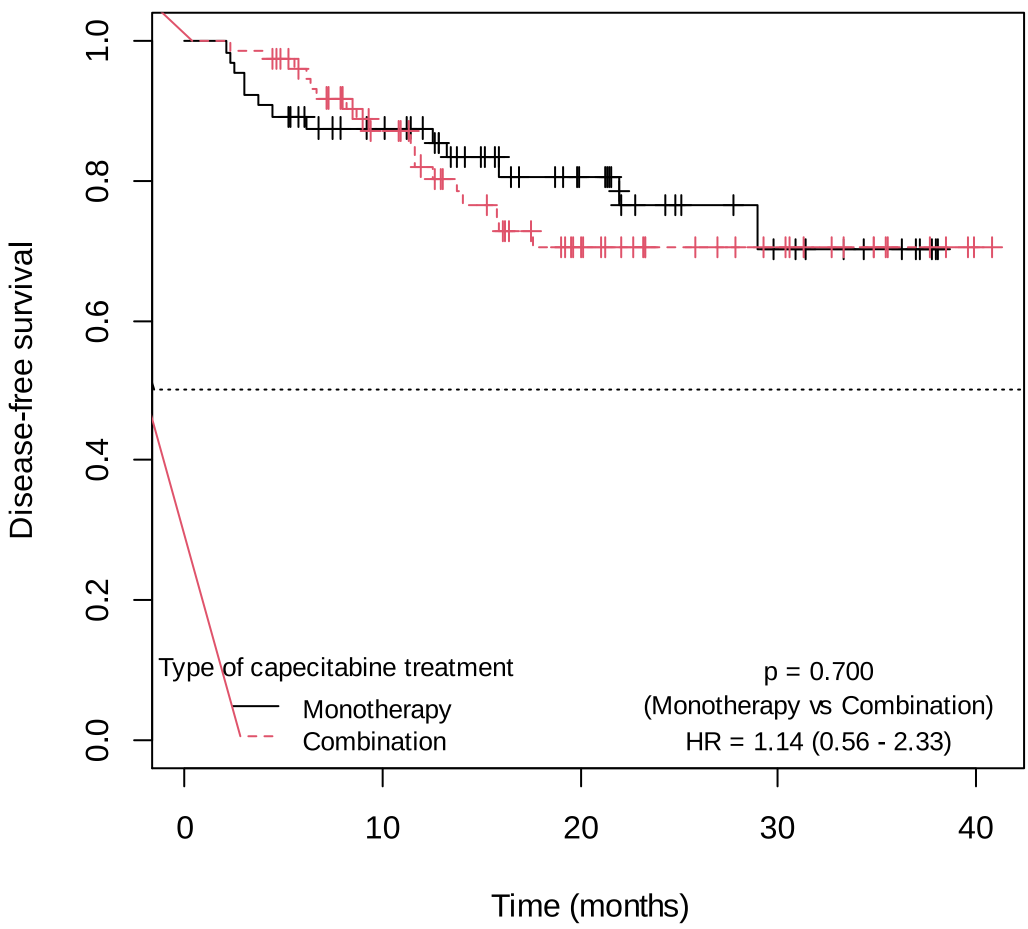

Figure S5. Kaplan-Meier survival curve for the association of DFS with type of adjuvant treatment

Table S10. Association of SNPs in capecitabine's pharmacodynamics with disease-free survival.

Gene	SNP	Genotype	Disease-free survival								
			N	Events	MST (m)	95% CI	p-value [a]	Ref. cat.	Bivariate Cox model		
									HR	95% CI	p-value
		CC	63	18	NA	NA - NA			1.00	-	
		CT	60	10	NA	NA - NA	0.300	CC	0.56	0.26 - 1.23	0.300
TYMS	rs2853741	TT	19	3	NA	NA - NA			0.52	0.15 - 1.80	
		C	123	28	NA	NA - NA	0.500	C	0.67	0.20 - 2.21	0.500
		T	79	13	NA	NA - NA	0.100	CC	0.56	0.27 - 1.14	0.100
		AA	80	21	NA	NA - NA			1.00	-	
		AG	46	7	NA	NA - NA	0.400	AA	0.56	0.23 - 1.32	0.400
	rs2790	GG	16	3	NA	29.00 - NA			0.65	0.19 - 2.18	
		A	126	28	NA	NA - NA	0.700	A	0.77	0.23 - 2.56	0.700
TYMS/ ENOSF1		G	62	10	NA	NA - NA	0.200	AA	0.58	0.27 - 1.24	0.200
		CC	56	12	NA	NA - NA			1.00	-	
		CT	60	16	NA	22 - NA	0.300	CC	1.34	0.63 - 2.84	0.200
	rs699517	TT	26	3	NA	NA - NA			0.50	0.14 - 1.77	
		C	116	28	NA	NA - NA	0.200	C	0.42	0.13 - 1.40	0.100
		T	86	19	NA	NA - NA	0.900	CC	1.06	0.51 - 2.18	0.900
		CC	24	8	NA	13.20 - NA			1.00	-	
		CT	62	17	NA	NA - NA	**0.040**	CC	0.87	0.37 - 2.03	**0.030**
ENOSF1	rs2612091	TT	56	6	NA	NA - NA			**0.31**	**0.10 - 0.90**	
		C	86	25	NA	NA - NA	**0.010**	C	0.34	0.14 - 0.83	**0.009**
		T	118	23	NA	NA - NA	0.200	CC	0.59	0.26 - 1.33	0.200
		GG	9	0	NA	NA - NA					
		GT	59	16	NA	29.00 - NA	0.200	-	-	-	0.080
	rs1801131	TT	74	15	NA	NA - NA					
		G	68	16	NA	NA - NA	0.600	G	0.81	0.40 - 1.65	0.600
		T	133	31	NA	NA - NA	0.100	-	-	-	0.040
MTHFR		AA	26	6	NA	NA - NA			1.00	-	
		AG	69	15	NA	NA - NA	1.000	AA	0.92	0.35 - 2.39	1.00
	rs1801133	GG	47	10	NA	29.00 - NA			0.87	0.31 - 2.40	
		A	95	21	NA	NA - NA	0.800	A	0.92	0.43 - 1.95	0.800
		G	116	25	NA	NA - NA	0.800	AA	0.90	0.36 - 2.20	0.800
		AA	50	12	NA	NA - NA			1.00	-	
		AG	74	15	NA	NA - NA	0.600	AA	0.68	0.31 - 1.45	0.600
	rs11615	GG	18	4	NA	NA - NA			0.82	0.26 - 2.54	
		A	124	27	NA	NA - NA	1.000	A	1.03	0.36 - 2.95	1.000
ERCC1		G	92	19	NA	NA - NA	0.300	AA	0.70	0.34 - 1.45	0.400
		AA	8	1	NA	NA - NA			1.00	-	
		AC	55	11	NA	NA - NA	0.600	AA	2.03	0.26 - 15.77	0.500
	rs3212986	CC	79	19	NA	NA - NA			2.59	0.34 - 19.44	
		A	63	12	NA	NA - NA	0.400	A	1.38	0.67 - 2.85	0.400
		C	134	30	NA	NA - NA	0.400	AA	2.35	0.32 - 17.31	0.300
		AA	9	3	NA	15.80 - NA			1.00	-	
		AG	61	8	NA	NA - NA	0.200	AA	0.37	0.10 - 1.43	0.200
	rs1799787	GG	72	20	NA	NA - NA			0.66	0.19 - 2.25	
		A	70	11	NA	NA - NA	0.300	A	1.46	0.69 - 3.05	0.300
		G	133	28	NA	NA - NA	0.300	AA	0.54	0.16 - 1.80	0.400
		GG	16	4	NA	15.80 - NA			1.00	-	
		GT	65	10	NA	NA - NA	0.400	GG	0.52	0.16 - 1.66	0.400
ERCC2	rs13181	TT	61	17	NA	29.00 - NA			0.84	0.28 - 2.52	
		G	81	14	NA	NA - NA	0.300	G	1.40	0.69 - 2.85	0.300
		T	126	27	NA	NA - NA	0.500	GG	0.68	0.23 - 1.97	0.500
		CC	70	18	NA	NA - NA			1.00	-	
		CT	60	9	NA	NA - NA	0.200	CC	0.61	0.27 - 1.36	0.300
	rs1799793	TT	12	4	NA	15.80 - NA			1.55	0.52 - 4.60	
		C	130	27	NA	NA - NA	0.200	C	1.87	0.65 - 5.39	0.300
		T	72	13	NA	NA - NA	0.400	CC	0.75	0.36 - 1.53	0.400
		CC	55	11	NA	NA - NA			1.00	-	
		CT	70	16	NA	NA - NA	0.800	CC	1.18	0.55 - 2.55	0.800
XRCC1	rs25487	TT	17	4	NA	22.00 - NA			1.36	0.43 - 4.28	
		C	125	27	NA	NA - NA	0.700	C	1.23	0.43 - 3.53	0.700
		T	87	20	NA	NA - NA	0.600	CC	1.21	0.58 - 2.54	0.600

Table S10. (Continue)

Gene	SNP	Genotype	Disease-free survival								
			N	Events	MST (m)	95% CI	p-value [a]	Ref. cat.	Bivariate Cox model		
									HR	95% CI	p-value
XRCC3	rs861539	AA	26	4	NA	NA - NA	0.500	AA	1.00	-	0.500
		AG	63	14	NA	NA - NA			1.52	0.50 - 4.65	
		GG	53	13	NA	29.00 - NA			1.85	0.60 - 5.70	
		A	89	18	NA	NA - NA	0.400	A	1.35	0.66 - 2.77	0.400
		G	116	27	NA	NA - NA	0.300	AA	1.67	0.58 - 4.78	0.300

m: months; SLM, median survival time; n: number of patients; NA: not available. HR: Hazard ratio, 95% CI: 95% confidence interval; Ref. cat.: reference category; SNP: single nucleotide polymorphisms.

a. Log-rank p-value.

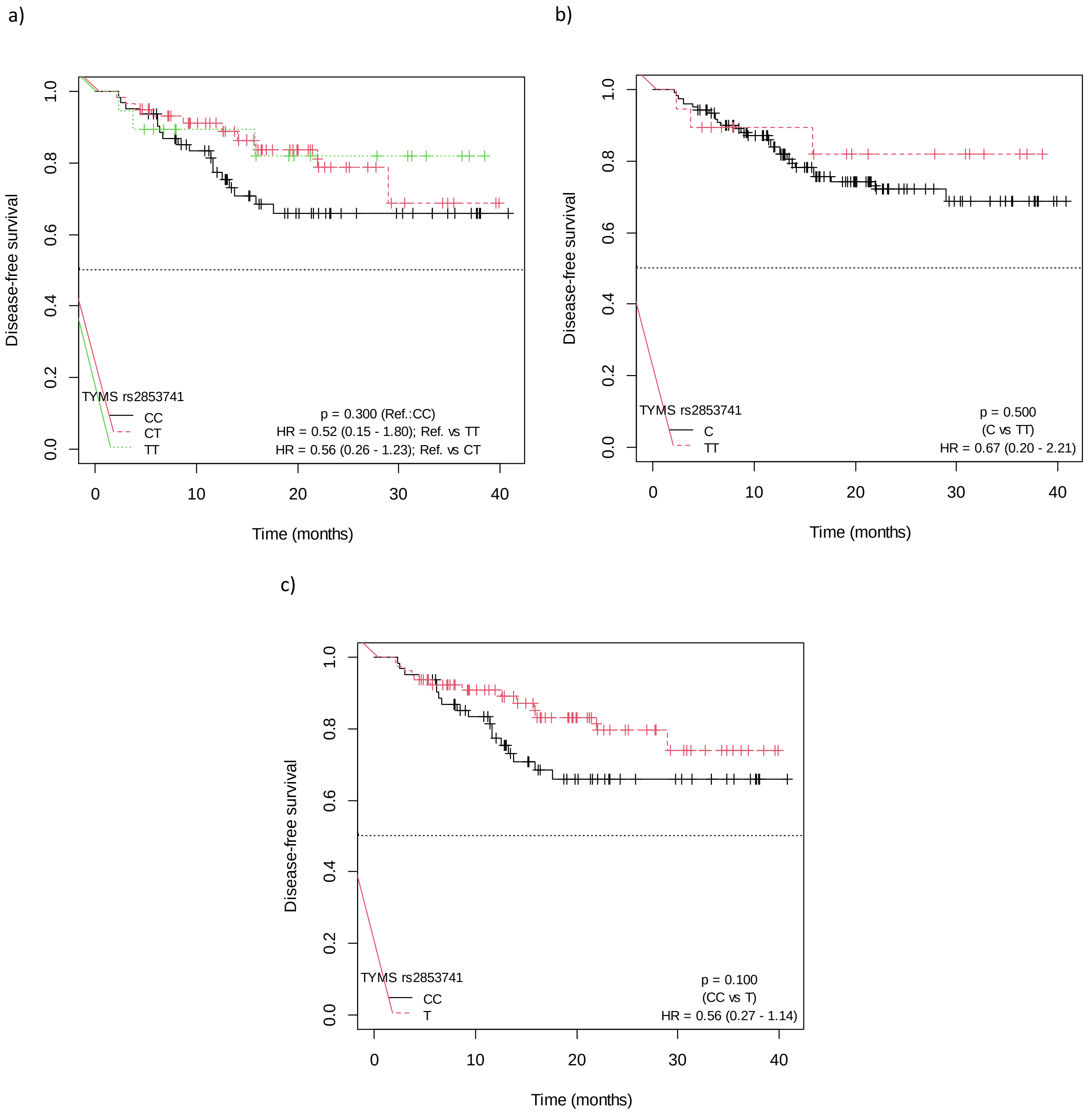

Figure S6. Kaplan-Meier curve for disease-free survival according to SNP *TYMS* rs2853741
a) *TYMS* rs2853741, b) *TYMS* rs2853741-C allele and c) *TYMS* rs2853741-T allele

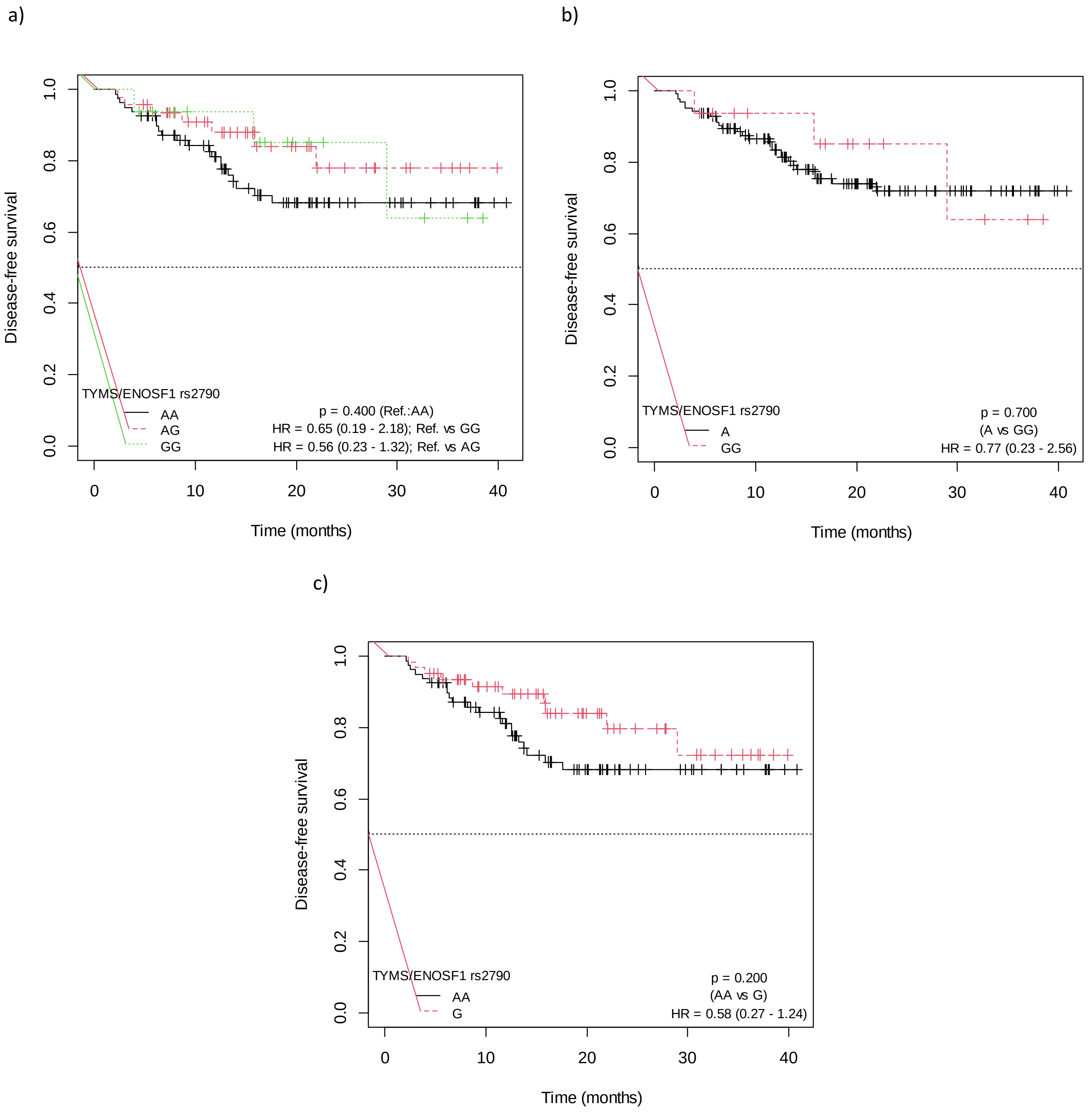

Figure S7. Kaplan-Meier curve for disease-free survival according to SNP *TYMS/ENOSF1* rs2790 a) *TYMS/ENOSF1* rs2790, b) *TYMS/ENOSF1* rs2790-A allele and c) *TYMS/ENOSF1* rs2790-G allele

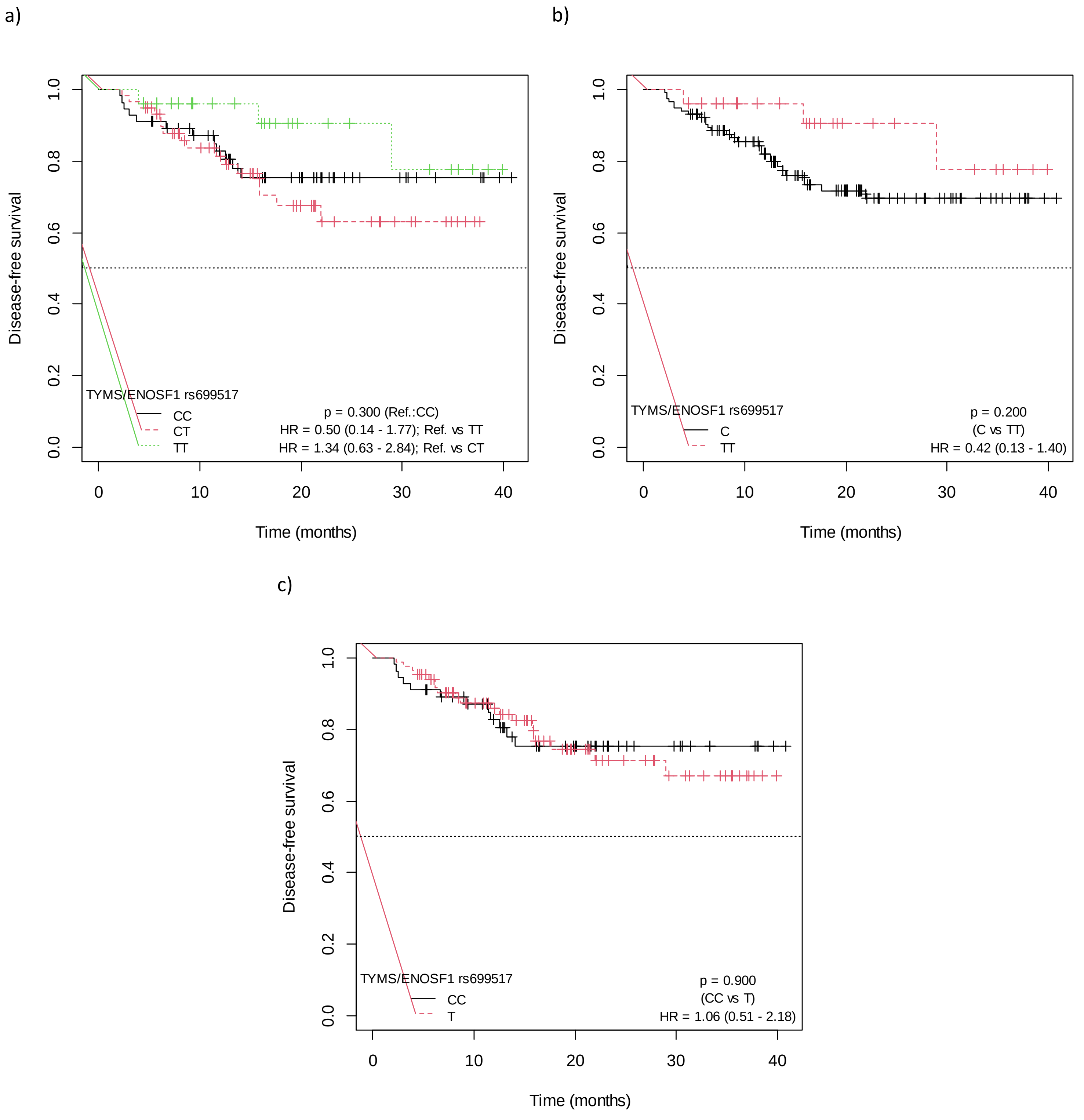

Figure S8. Kaplan-Meier curve for disease-free survival according to SNP *TYMS/ENOSF1* rs699517 a) *TYMS/ENOSF1* rs699517, b) *TYMS/ENOSF1* rs699517-C allele and c) *TYMS/ENOSF1* rs699517-T allele

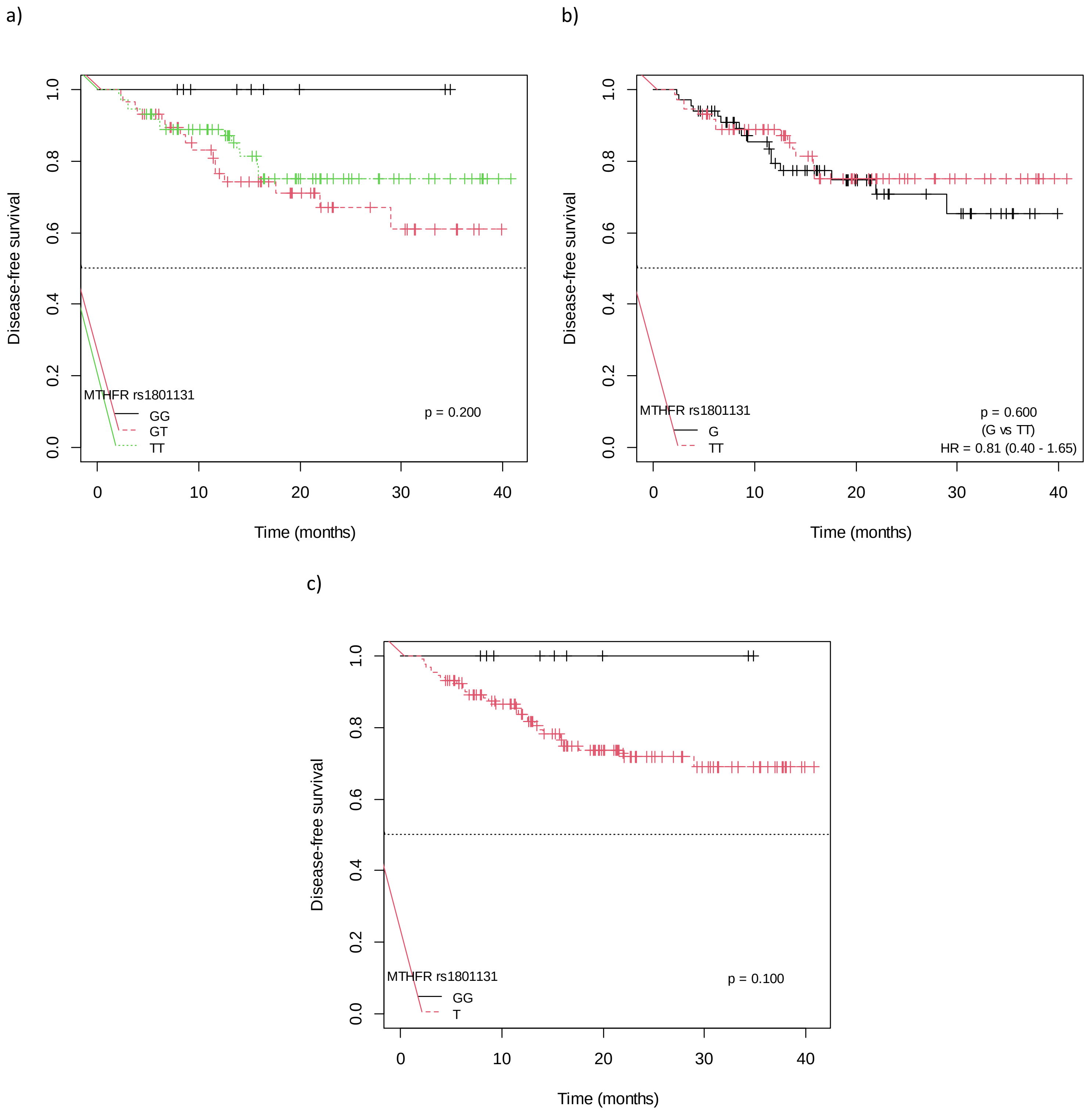

Figure S9. Kaplan-Meier curve for disease-free survival according to SNP *MTHFR* rs1801131 a) *MTHFR* rs1801131, b) *MTHFR* rs1801131-G allele and c) *MTHFR* rs1801131-T allele

a) b)

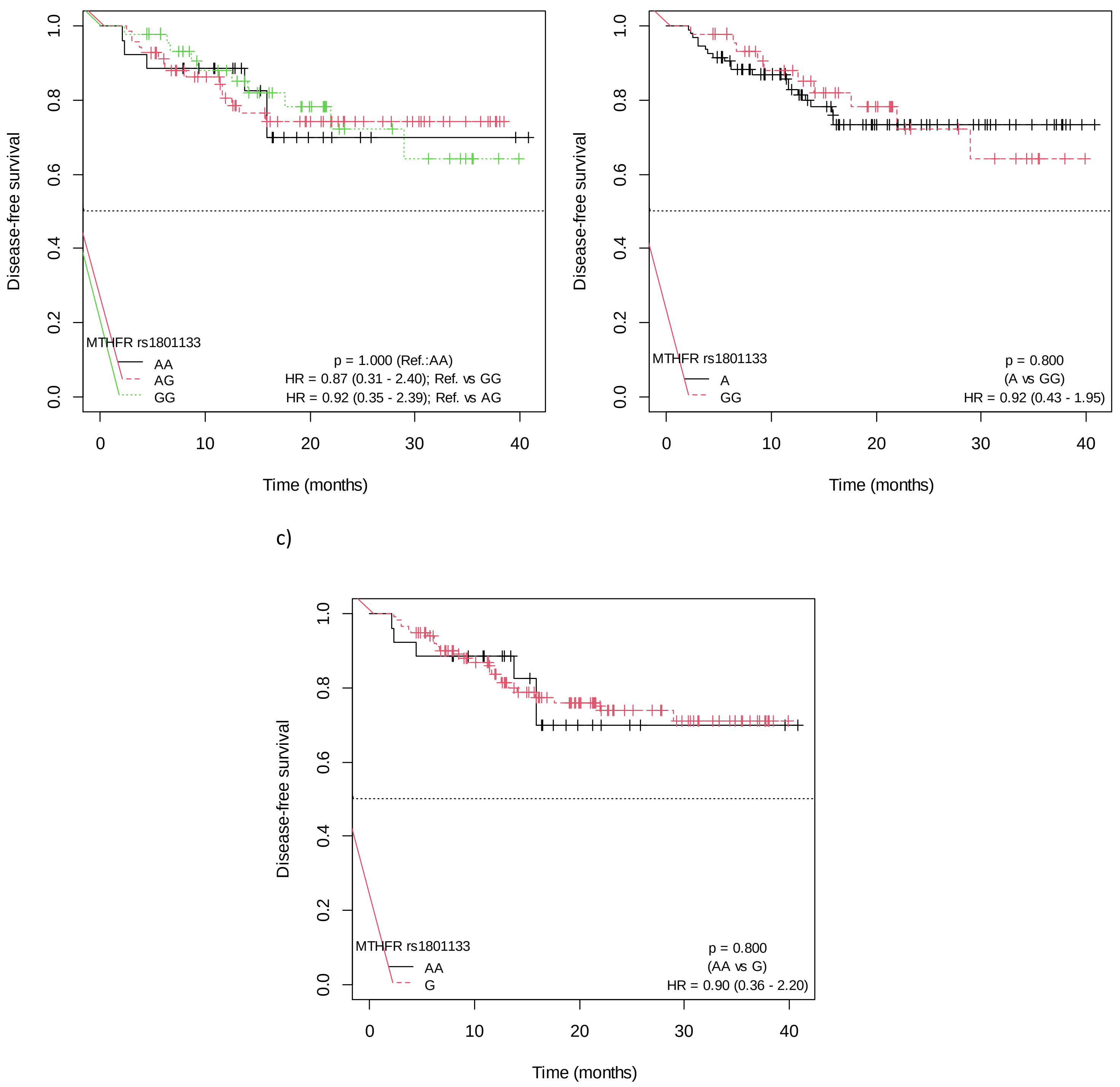

Figure S10. Kaplan-Meier curve for disease-free survival according to SNP *MTHFR* rs1801133 a) *MTHFR* rs1801133, b) *MTHFR* rs1801133-A allele and c) *MTHFR* rs1801131-G allele

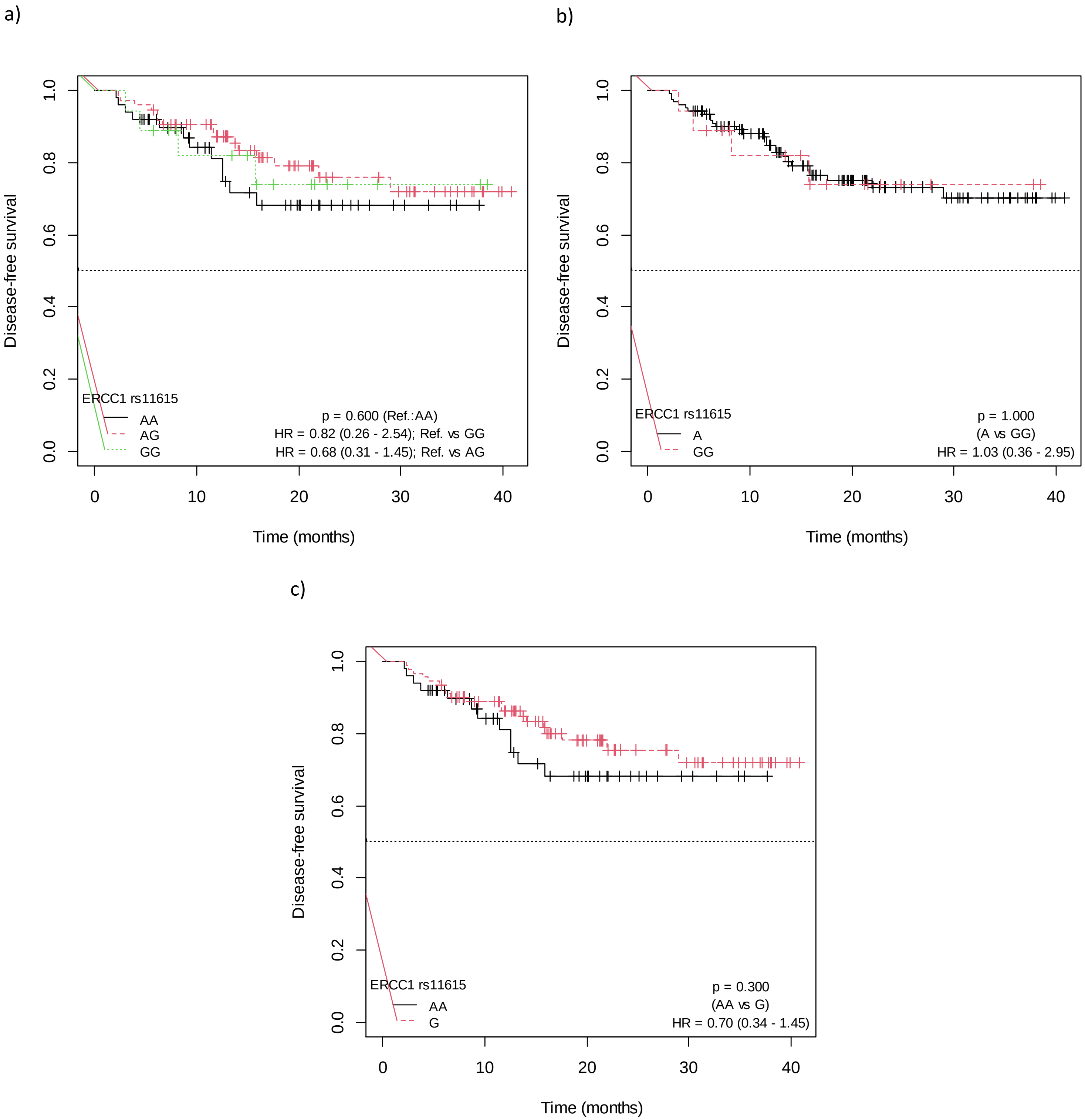

Figure S11. Kaplan-Meier curve for disease-free survival according to SNP *ERCC1* rs11615

a) *ERCC1* rs11615, b) *ERCC1* rs11615-A allele and c) *ERCC1* rs11615-G allele

a)

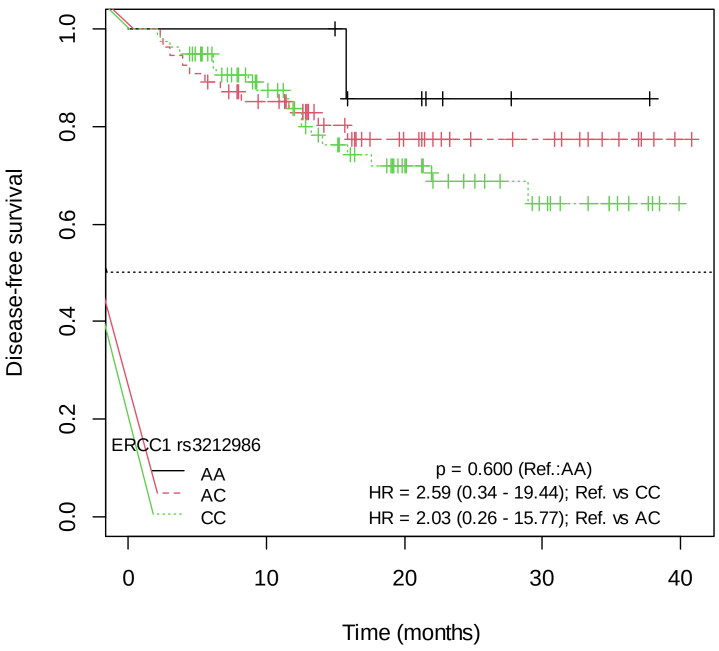

b)

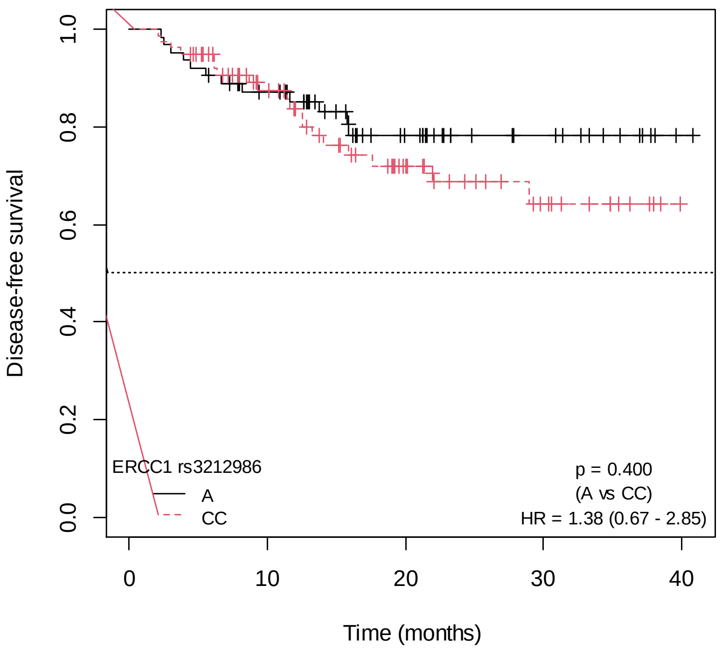

c)

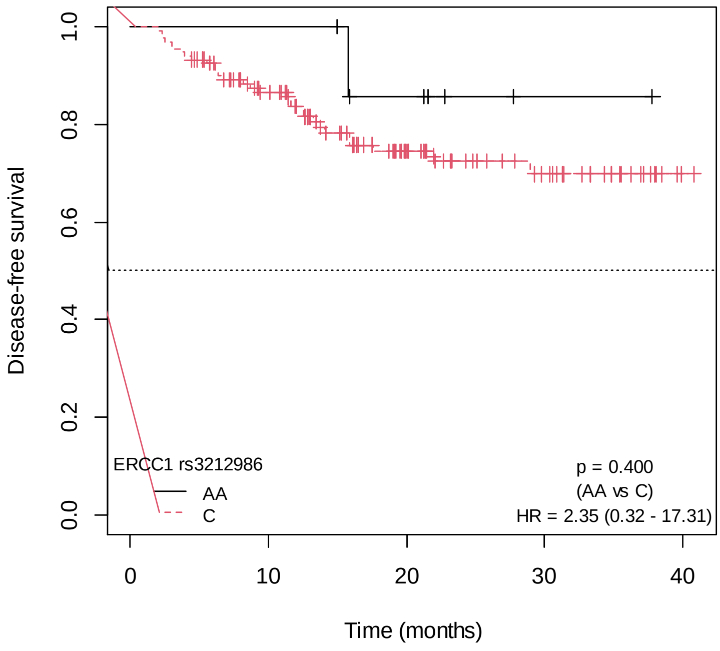

Figure S12. Kaplan-Meier curve for disease-free survival according to SNP *ERCC1* rs3212986
a) *ERCC1* rs3212986, b) *ERCC1* rs3212986-A allele and c) *ERCC1* rs3212986-C allele

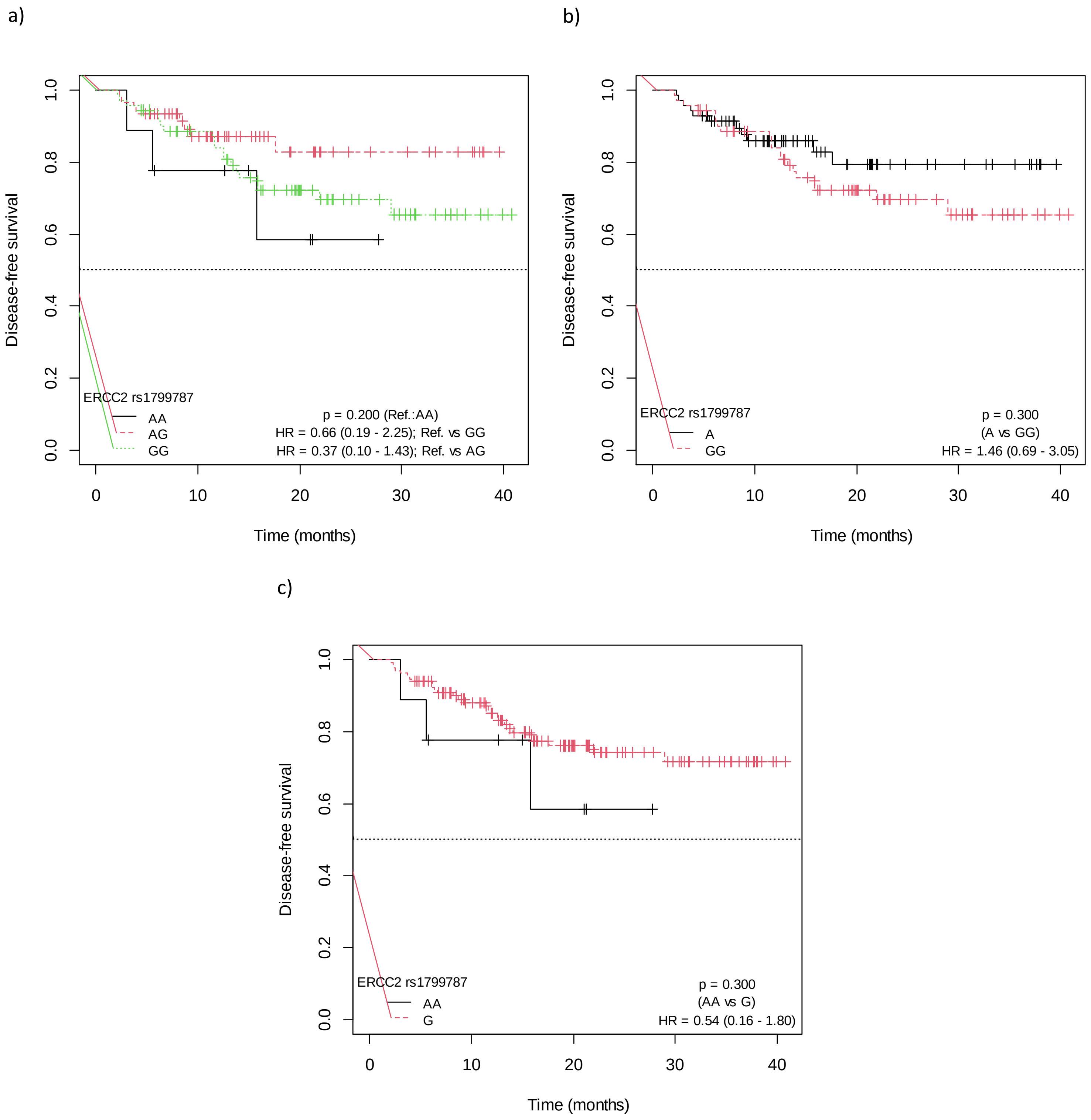

Figure S13. Kaplan-Meier curve for disease-free survival according to SNP *ERCC2 rs1799787*

a) *ERCC2 rs1799787*, b) *ERCC2 rs1799787-A* allele and c) *ERCC2 rs1799787-G* allele

a)

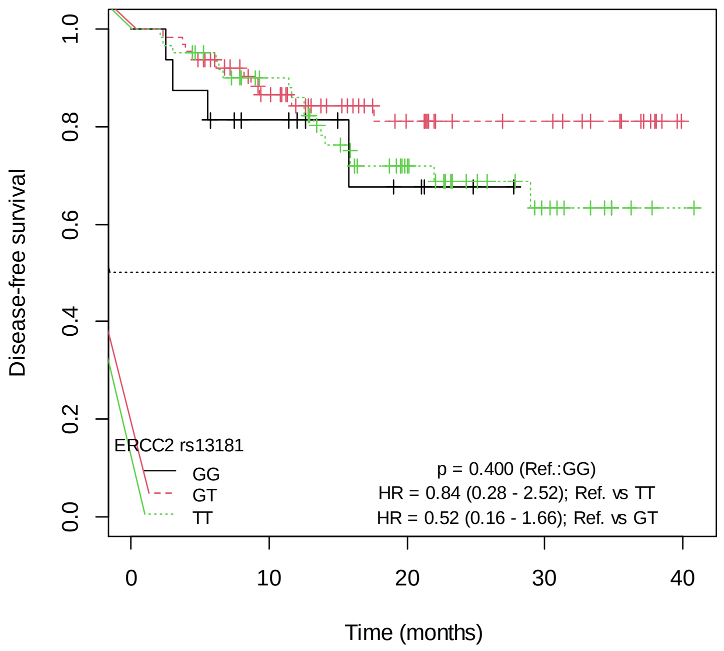

b)

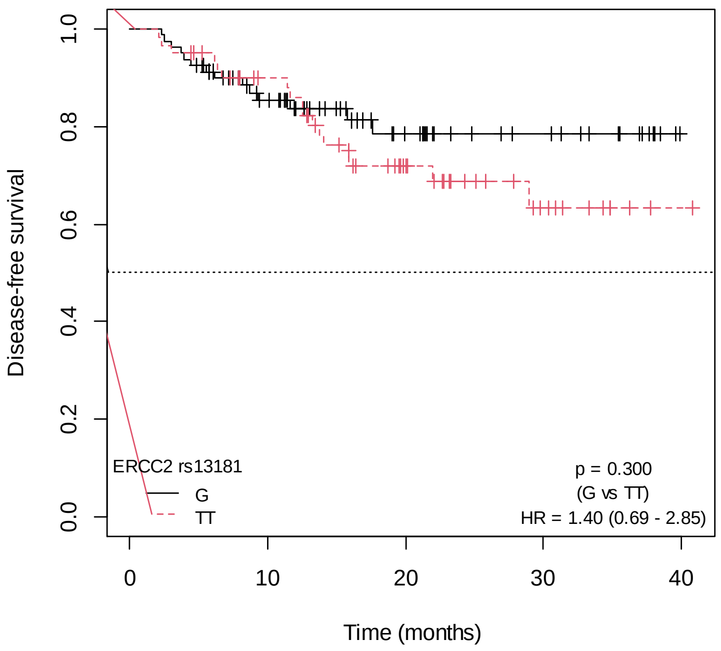

c)

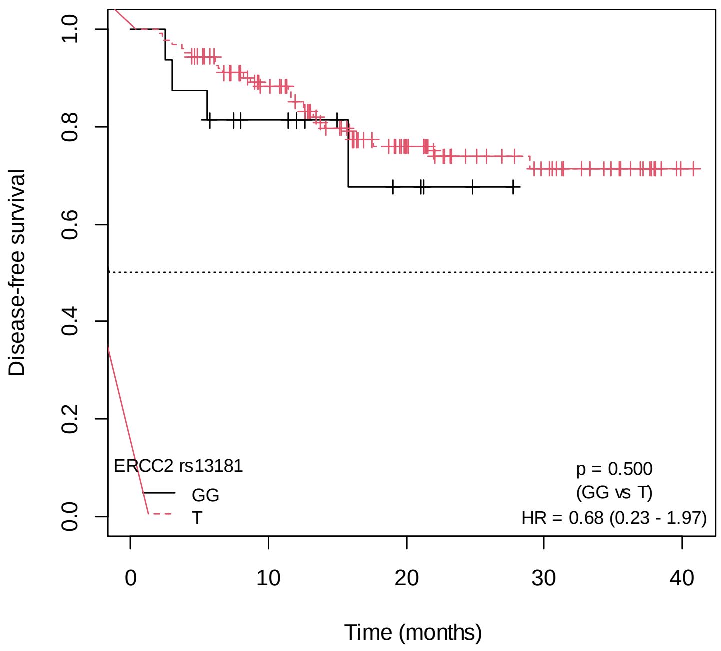

Figure S14. Kaplan-Meier curve for disease-free survival according to SNP *ERCC2* rs13181

a) *ERCC2* rs13181, b) *ERCC2* rs13181-G allele and c) *ERCC2* rs13181-T allele

a)

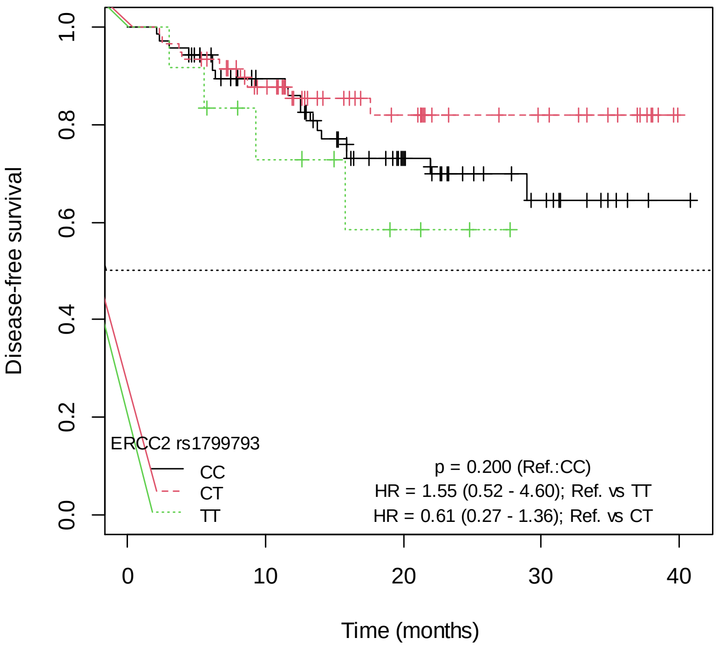

b)

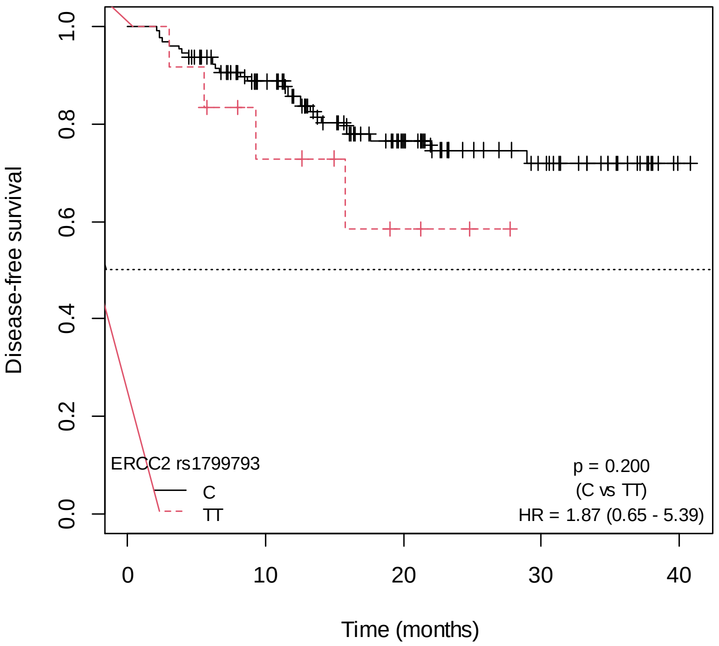

c)

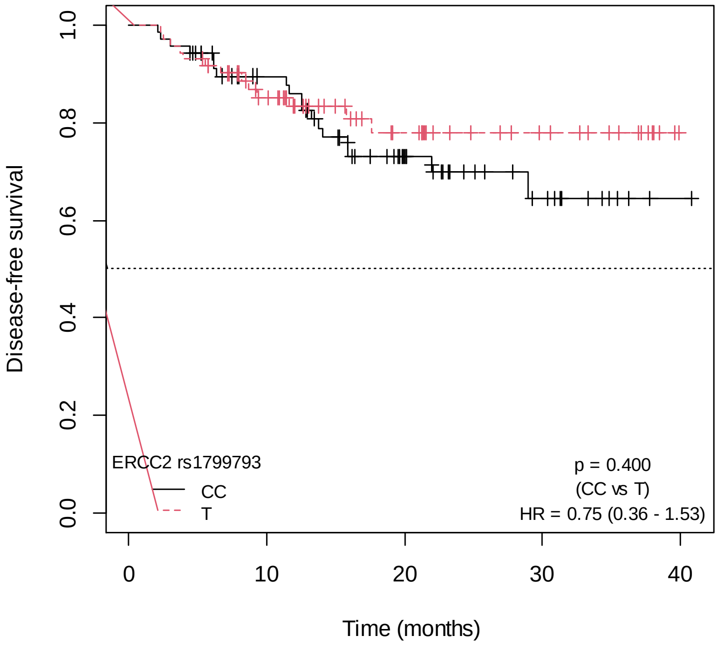

Figure S15. Kaplan-Meier curve for disease-free survival according to SNP *ERCC2* rs1799793
a) *ERCC2* rs1799793, b) *ERCC2* rs1799793-C allele and c) *ERCC2* rs1799793-T allele

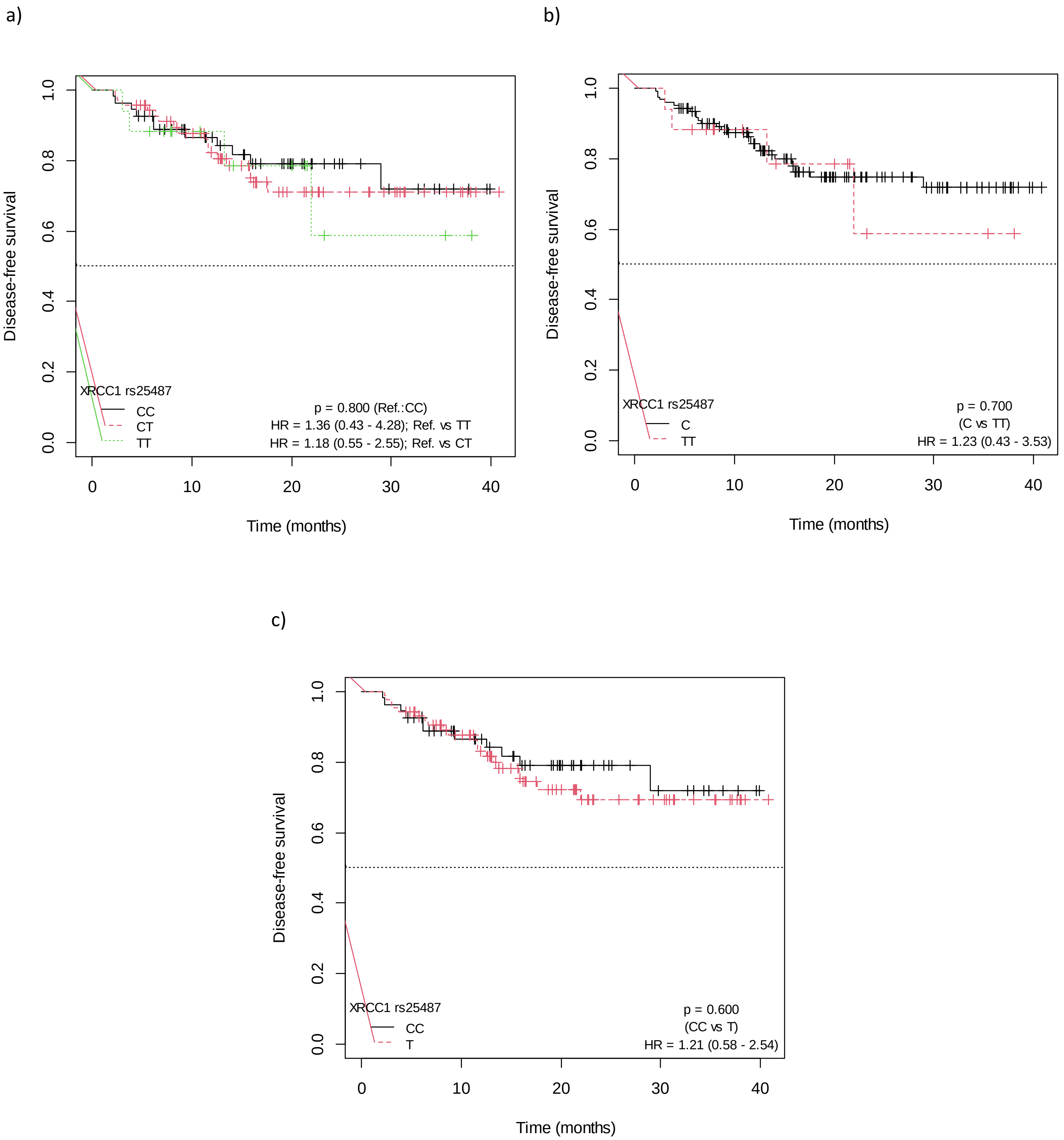

Figure S16. Kaplan-Meier curve for disease-free survival according to SNP *XRCC1* rs25487

a) *XRCC1* rs25487, b) *XRCC1* rs25487-C allele and c) *XRCC1* rs25487-T allele

a)

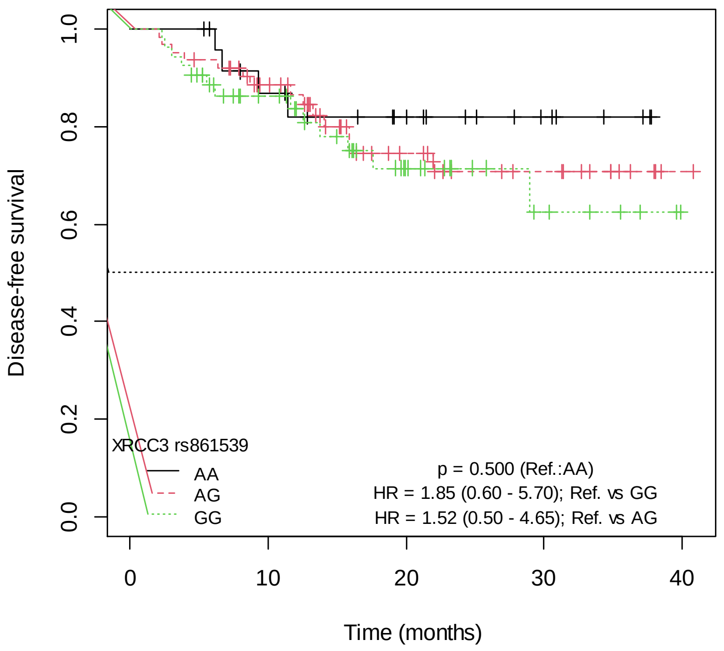

b)

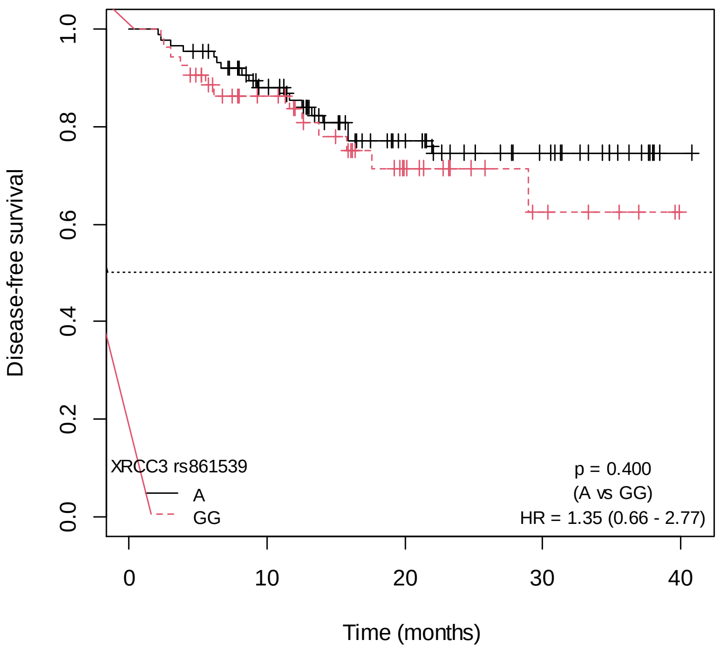

c)

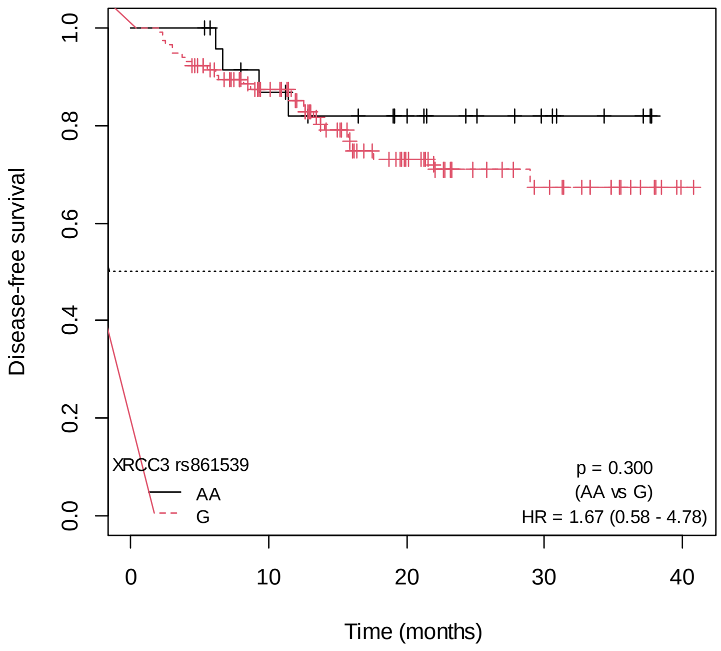

Figure S17. Kaplan-Meier curve for disease-free survival according to SNP *XRCC3* rs861539
a) *XRCC3* rs861539, b) *XRCC3* rs861539-A allele and c) *XRCC3* rs861539-G allele

Table S11. *MTHFR* haplotype effects estimation on disease-free survival.

H	*MTHFR* rs1801131	*MTHFR* rs1801133	Frequency	HR (95%CI)	p-value
0	T	A	0.426	1.00	-
1	T	G	0.303	0.93 (0.48 - 1.79)	0.833
2	G	G	0.271	0.93 (0.46 - 1.89)	0.852
Goodness-of-fit: -2x Log-likelihood (with covariates) = 285.724688					
Goodness-of-fit: -2 x Log-likelihood (without covariates) =285.795061					
df. = 2 ; model p-value: 0.965					

df: degrees of freedom; H: haplotype; HR: Hazard ratio; 95%CI: 95% confidence interval.

Table S12. *ERCC2* haplotype effects estimation on disease-free survival.

H	*ERCC2* rs179787	*ERCC2* rs13181	Frequency	HR (95%CI)	p-value
0	G	T	0.659	1.00	-
1	A	G	0.278	0.84 (0.49 - 1.46)	0.556
2	G	G	0.063	0.97 (0.33 - 2.79)	0.958
Goodness-of-fit: -2x Log-likelihood (with covariates) = 285.509557					
Goodness-of-fit: -2 x Log-likelihood (without covariates) = 285.795061					
df. = 2 ; model p-value: 0.867					

df: degrees of freedom; H: haplotype; HR: Hazard ratio; 95%CI: 95% confidence interval.

Table S13. *ERCC1* haplotype effects estimation on disease-free survival.

H	*ERCC1* rs3212986	*ERCC1* rs11615	Frequency	HR (95%CI)	p-value
0	C	A	0.600	1.00	-
1	A	G	0.238	0.76 (0.40 - 1.44)	0.400
2	C	G	0.150	0.96 (0.44 - 2.09)	0.930
3	A	A	0.012	-	-
Goodness-of-fit: -2x Log-likelihood (with covariates) = 285.014061					
Goodness-of-fit: -2 x Log-likelihood (without covariates) = 285.795061					
df. = 2; model p-value: 0.676					

df: degrees of freedom; H: haplotype; HR: Hazard ratio; 95%CI: 95% confidence interval.
